# Modifications of Blood Molecular Components after Treatment with Low Ozone Concentrations

**DOI:** 10.3390/ijms242417175

**Published:** 2023-12-06

**Authors:** Chiara Rita Inguscio, Barbara Cisterna, Flavia Carton, Elettra Barberis, Marcello Manfredi, Manuela Malatesta

**Affiliations:** 1 Department of Neurosciences, Biomedicine and Movement Sciences, University of Verona, Strada Le Grazie 8, 37134 Verona, Italy; chiararita.inguscio@univr.it (C.R.I.); barbara.cisterna@univr.it (B.C.); flavia.carton@univr.it (F.C.); 2 Department of Sciences and Technological Innovation, University of Piemonte Orientale, Viale T. Michel 11, 15121 Alessandria, Italy; elettra.barberis@uniupo.it; 3 Center for Translational Research on Autoimmune and Allergic Diseases, University of Piemonte Orientale, Corso Trieste 15/A, 28100 Novara, Italy; marcello.manfredi@uniupo.it; 4 Department of Translational Medicine, University of Piemonte Orientale, Via Solaroli 17, 28100 Novara, Italy

**Keywords:** low-dose ozone, oxidative stress, antioxidant response, antioxidant capacity, interleukins, metabolomics, haemogas

## Abstract

The ex vivo treatment of a limited volume of blood with gaseous oxygen–ozone (O_2_–O_3_) mixtures and its rapid reinfusion into the patient is a widespread medical procedure. O_3_ instantly reacts with the blood’s antioxidant systems, disappearing before reinfusion, although the molecules formed act as messengers in the organism, inducing multiple antioxidant and anti-inflammatory responses. An appropriate dose of O_3_ is obviously essential to ensure both safety and therapeutic efficacy, and in recent years, the low-dose O_3_ concept has led to a significant reduction in the administered O_3_ concentrations. However, the molecular events triggered by such low concentrations in the blood still need to be fully elucidated. In this basic study, we analysed the molecular modifications induced ex vivo in sheep blood by 5 and 10 µg O_3_/mL O_2_ by means of a powerful metabolomics analysis in association with haemogas, light microscopy and bioanalytical assays. This combined approach revealed increased oxygenation and an increased antioxidant capacity in the O_3_-treated blood, which accorded with the literature. Moreover, original information was obtained on the impact of these low O_3_ concentrations on the metabolic pathways of amino acids, carbohydrates, lipids and nucleotides, with the modified metabolites being mostly involved in the preservation of the oxidant–antioxidant balance and in energy production.

## 1. Introduction

Ozone (O_3_) is an unstable gas occurring in the Earth’s atmosphere, where it naturally forms from oxygen (O_2_) due to the action of ultraviolet light and electrical discharges and then rapidly decomposes into O_2_. Its high oxidation power makes O_3_ harmful to organisms; however, if appropriately applied, it may be used as a therapeutic agent. The beneficial properties of O_3_ have been known since the 18th century and, starting from the 19th century, its use in medicine has progressively expanded for the treatment of an increasing number of diseases through different administration routes [1].

The ex vivo treatment of a limited volume (100–200 mL) of whole blood with gaseous O_2_–O_3_ mixtures and rapid reinfusion into the patient via the venous route is a widespread medical procedure first described by the Austrian physician H. Wolff in the 1970s [2]. This procedure was initially applied on an empirical basis but, starting from the 1990s, with the advent of O_3_ generators able to produce photometrically-measured O_3_ concentrations in real time and in a given gas volume, the administration protocols were refined via precisely determining the actually administered O_3_ dosages. Notably, this also allowed conducting accurate scientific investigations into the effect of O_3_ on blood components, thus unveiling the basic molecular mechanisms involved in its therapeutic potential [3,4,5].

Scientific research has demonstrated that, if administered to blood at appropriate (low) doses, the deleterious oxidising effect of O_3_ can be neutralised by the plasmatic antioxidant factors, such as uric acid, ascorbic acid, glutathione (GSH), albumin and lipophilic molecules, as well as by the great reservoir of GSH and other antioxidant enzymes located in the erythrocytes [4,6]. In the blood, the O_2_–O_3_ mixture immediately dissolves into the water of the plasma: O_2_ fully oxygenates haemoglobin while O_3_ instantly reacts with hydrophilic antioxidants and polyunsaturated fatty acids (PUFA), giving rise to the formation of H_2_O_2_ and a variety of lipid oxidation products (LOPs) [7,8,9]. Since these reactions take place in a few seconds, the O_3_ present in the administered gas mixture disappears in 2–5 min from the blood contained in the bottle before its reinfusion, thus never entering circulation after blood reinfusion. However, as a result of these reactions, O_3_ causes a small and transient depletion of antioxidants and a plasmatic increase in reactive oxygen species (ROS) and LOPs [10], which after blood reinfusion act as messengers in the whole organism, activating biochemical and immunological pathways and initiating cascades of biological events (e.g., production of growth factors and cytokines, upregulation of many antioxidant enzymes) in various tissues and organs (exhaustive reviews in [3,4,6,11]). Therefore, the therapeutic potential of blood ozonation observed in multiple diseases (recent publications in [11,12,13,14,15,16,17]) relies on biological events triggered by O_3_ ex vivo in the drawn blood and then accomplished in the organism by the O_3_ derivatives generated as a physiological response to the mild oxidative stress [3,4,5,18].

An appropriate dose of gaseous O_3_ is essential to ensure both safety and therapeutic efficacy because it must not exceed the blood antioxidant potential but, at the same time, it must generate enough molecular messengers to induce beneficial biological effects. A concentration window of 20–80 µg O_3_/mL O_2_ was identified as suitable in the 1990s [19,20] but, during the last decade, the clinical experience and the scientific evidence led to a significant reduction in the concentration of the administered O_3_ while maintaining its therapeutic efficacy, according to the low-dose O_3_ concept [21,22], although the molecular effects of such low concentrations on blood still need to be fully elucidated.

In light of this, in order to provide additional knowledge of the molecular events triggered by O_3_ in blood, we conducted a basic study analysing the molecular modifications induced ex vivo by gaseous O_2_–O_3_ mixtures at O_3_ concentrations of 5 µg and 10 µg. To ensure highly controlled experimental conditions when administering such low O_3_ concentrations, a next-generation O_3_ generator was used and a powerful metabolomics analysis was applied in association with haemogas analysis, light microscopy and bioanalytical assays. 

## 2. Results

### 2.1. Haemogas Analysis

**Figure 1:**
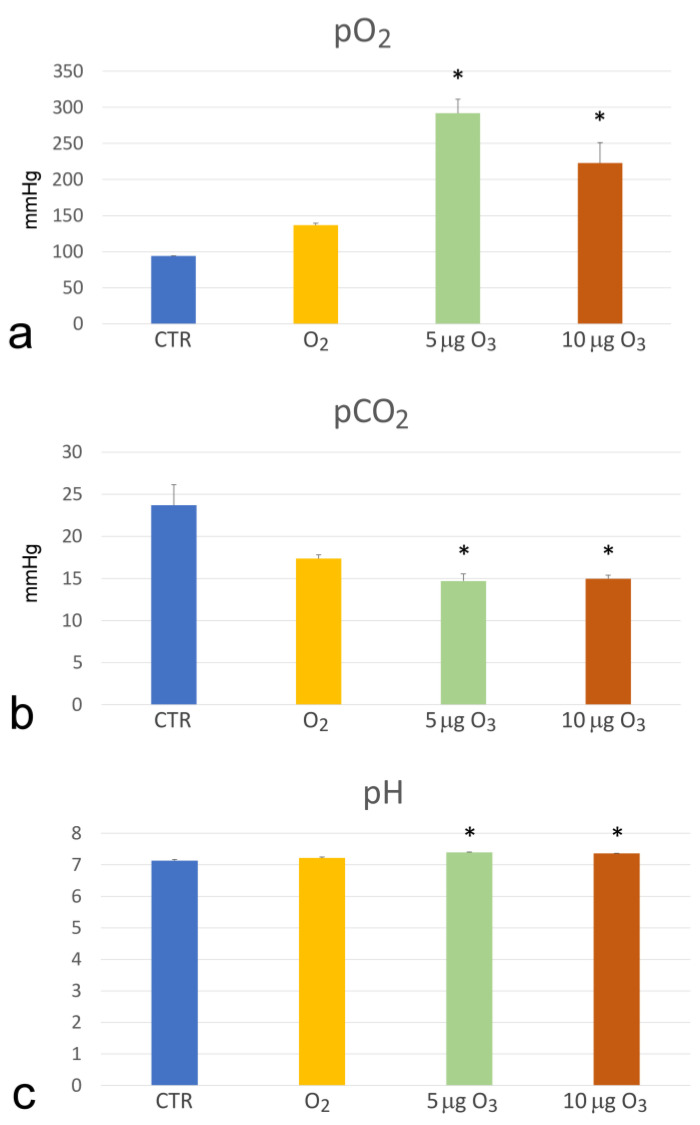
Mean value ± SE of pO_2_ (**a**), pCO_2_ (**b**) and pH (**c**) in the blood samples after gas treatment (*n* = 3). The asterisk (*) indicates a significant difference in comparison to the control (CTR).

The haemogas analysis showed that both 5 μg O_3_ and 10 μg O_3_ induced a significant increase in pO_2_ (*p* = 0.036 and *p* = 0.027, respectively) (Figure 1a) and pH (*p* = 0.028 and *p* = 0.026, respectively) (Figure 1c) compared to the controls. On the contrary, a decrease in pCO_2_ was observed in the 5 μg O_3_- and 10 μg O_3_-treated samples in comparison to the controls (*p* = 0.028 for both concentrations) (Figure 1b).

### 2.2. Haemolysis

**Figure 2:**
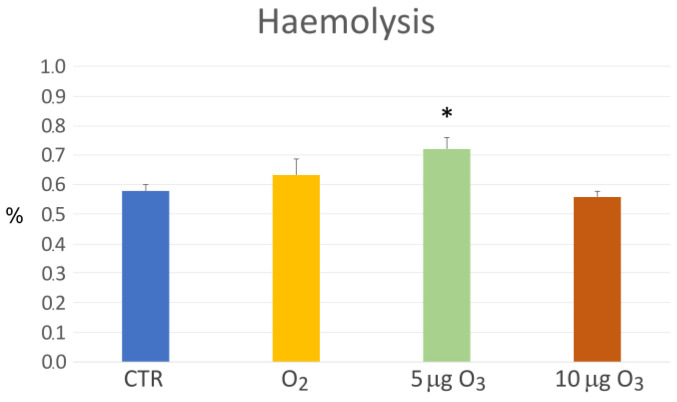
Mean value ± SE of the haemolysis rate in the blood samples after gas treatment (*n* = 3). The asterisk (*) indicates a significant difference in comparison to the control (CTR).

The blood haemolysis assessment, carried out via a colorimetric assay for the haemoglobin evaluation, revealed no statistically significant difference in the O_2_- and 10 μg O_3_-treated samples in comparison to the control values, while a significant increase in haemolysis was found in the blood sample treated with 5 μg O_3_ (*p* = 0.008) (Figure 2). 

**Figure 3:**
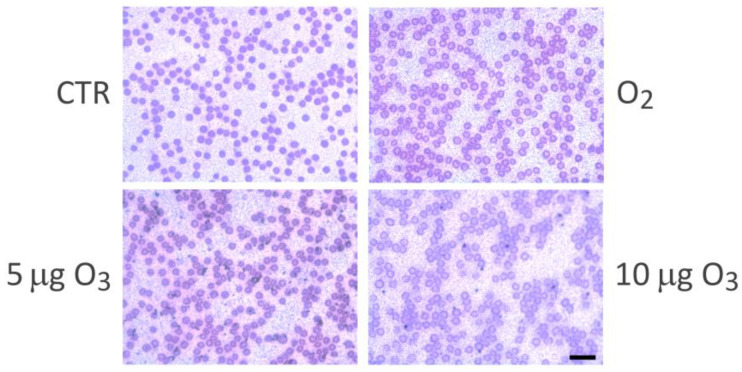
The blood smears collected from the control (CTR) and gas-treated samples show well-preserved erythrocytes. Bar = 10 µm.

The blood smears showed in all the samples well-preserved erythrocytes with no evident morphological sign of damage (Figure 3).

### 2.3. Total Antioxidant Capacity

**Figure 4:**
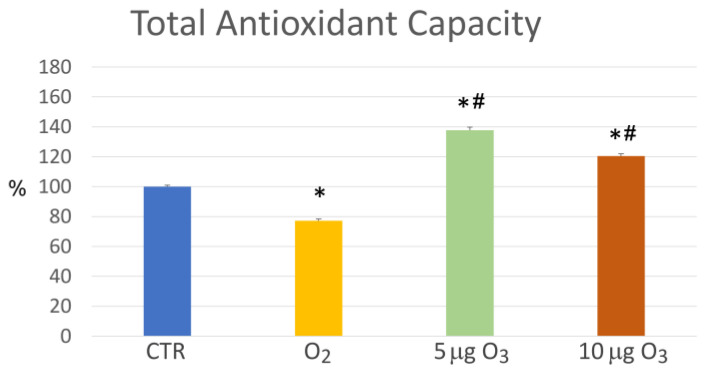
Mean value ± SE of the total antioxidant capacity of the blood samples after gas treatment (*n* = 3). The asterisk (*) indicates a significant difference in comparison to the control (CTR); the symbol # indicates a significant difference in comparison to O_2_.

The total antioxidant capacity, evaluated via a colorimetric assay for the measurement of the antioxidant proteins and/or small molecules, significantly increased in the 5 μg O_3_- and 10 μg O_3_-treated blood samples in comparison to the controls (*p* = 0.004 for both concentrations); conversely, O_2_ significantly reduced the antioxidant capacity in comparison to the control (*p* = 0.011) (Figure 4). In addition, the antioxidant capacity of the 5 μg O_3_- and 10 μg O_3_-treated samples was significantly higher than in the O_2_-treated samples (*p* = 0.003 and *p* = 0.004, respectively).

### 2.4. Interleukins

**Figure 5:**
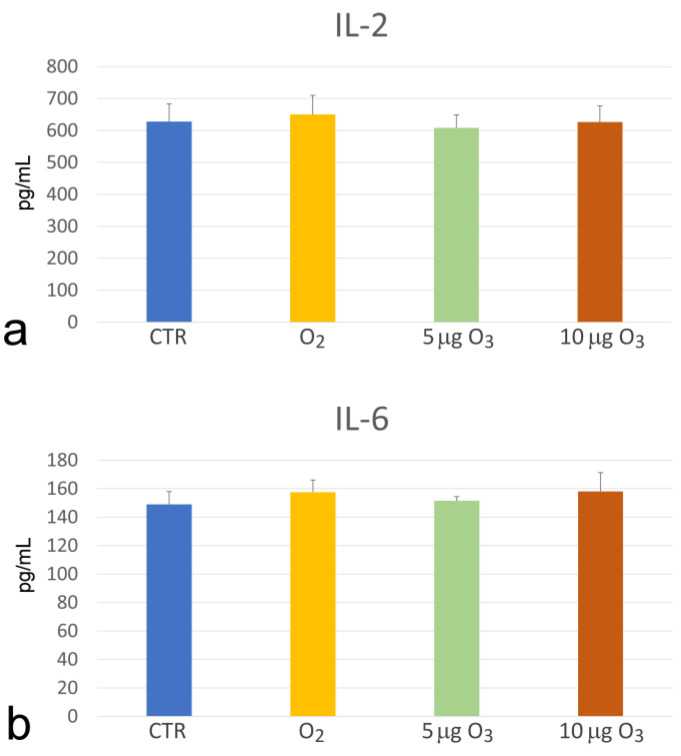
Mean value ± SE of the IL-2 (**a**) and IL-6 (**b**) amounts in the blood samples after gas treatment (*n* = 3). No significant difference was found among the samples.

The amounts of the interleukins IL-2 and IL-6 were found to be unchanged by any treatment in comparison to the control samples (Figure 5). 

### 2.5. Metabolomics 

**Figure 6:**
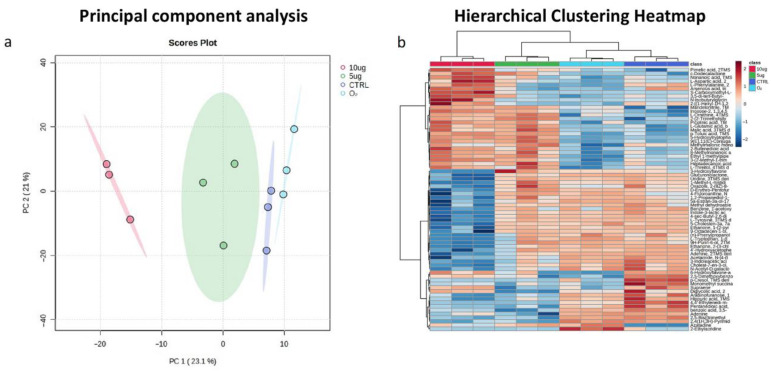
Metabolomic signature associated with O_3_ treatment. Principal component analysis (**a**) and hierarchical clustering heatmap (**b**) performed on the metabolomic data of blood samples treated with 10 μg O_3_ (red), 5 μg O_3_ (green), O_2_ (light blue) and untreated (blue).

Untargeted metabolomic analysis showed that both the 5 μg O_3_ and 10 μg O_3_ treatments induced significant molecular changes compared to the controls. Principal component analysis (PCA) and the hierarchical clustering heatmap (Figure 6) clearly highlighted the presence of a metabolic signature associated with O_3_ treatment. In addition, both PCA and clustering analysis showed the high reproducibility of the treatment, since all the resultant replicates were well grouped together.

A total of 572 metabolites were identified (Table S1). Since O_3_ was administered as an O_2_–O_3_ mixture, the metabolites showing a difference in comparison to the control after treatment with pure O_2_ were not considered. 

In comparison to the control, 25 metabolites showed statistically significant modifications after treatment with 5 μg O_3_, and 89 metabolites after treatment with 10 µg O_3_; 31 metabolites showed a statistical difference with the control after treatment with both 5 μg O_3_ and 10 μg O_3_. Among these metabolites, only those showing a fold change ≥ 1.3 or ≤0.769 were considered. Moreover, metabolites corresponding to exposomes or specific food components were excluded. 

As the final result of this selection process, a list of molecules involved in protein, carbohydrate, lipid and nucleotide metabolism was obtained: some of them underwent a decrease while others increased (Table 1).

## 3. Discussion

The molecular modifications observed in the whole blood exposed to 5 μg O_3_ or 10 μg O_3_ demonstrate that these low O_3_ concentrations are able to significantly act on multiple chemical features and metabolic pathways. 

### 3.1. The Impact on Blood Oxygenation and Haemolysis

According to the well-known effects of blood ozonation, pO_2_ increased in both the O_3_-treated blood samples [23,24,25], whereas pCO_2_ decreased [25]. The increase in the pH value in the O_3_-treated samples is consistent with the concomitant increase in pO_2_. The reason for the higher pO_2_ value observed in the O_3_-treated samples in comparison to the O_2_-treated samples remains unclear. However, it should be underlined that the hyperoxygenation of ozonated blood occurring in the bottle is considered clinically irrelevant because this limited amount of blood (100–200 mL) is reinfused via the venous route during the following 15–20 min and is heavily diluted in the venous blood, which has a pO_2_ of about 40 mmHg, so that the final venous pO_2_ is hardly modified [3,4,5,24].

A limited but statistically significant increase in haemolysis was found in the blood samples treated with 5 µg O_3_, in the absence of microscopically detectable damage to the erythrocytes. Accordingly, a slight increase in haemolysis has been frequently reported after O_3_ treatment and has been always considered negligible [10,20,23,24,26]. 

### 3.2. The Increase in Blood Antioxidant Capacity

The increase in the antioxidant capacity has been considered for decades the rationale behind the therapeutic efficacy of blood ozonation [5,19], and the evidence in our O_3_-treated blood samples is consistent with previous results obtained in different experimental models. It has been reported that the treatment of blood with therapeutic doses of O_3_, after causing an initial slight and transient decrease in the antioxidant capacity of the plasma (fully reconstituted within a few minutes) [5], gives rise to a prompt plasmatic and cellular antioxidant response [4,18] that is likely responsible for the increased antioxidant capacity in our O_3_-treated samples. In particular, it has already been experimentally demonstrated that an O_3_ concentration of 10 μg is able to stimulate an antioxidant cytoprotective response through the activation of the Keap1-dependent nuclear factor erythroid 2-related factor 2 (Nrf2) pathway [27,28,29,30,31,32,33,34,35]. Interestingly, in the present study, we found that also the very low concentration of 5 μg O_3_ is able to induce a significant increase in the antioxidant capacity. On the other hand, pure O_2_ not only is unable to increase the antioxidant capacity but even induces its significant decrease, demonstrating that the O_2_-derived oxidative stress cannot activate an antioxidant response as low O_3_ doses actually do.

### 3.3. The Unchanged Levels of IL-2 and IL-6

Under our experimental conditions, O_3_ treatment did not modify the plasmatic amounts of IL-2 and IL-6, both characterized by a wide range of actions in the immune response. The literature data on the effect of therapeutic doses of O_3_ on cytokine secretion are heterogeneous: some studies described a stimulating action on leukocytes with increased IL-2 and IL-6 secretion [10,33,36], whereas others reported unchanged levels of IL-2 and IL-6 [37]. These discrepancies could be related to the different experimental conditions used, such as the O_3_ concentration, the treatment in vitro or in vivo, and the detection techniques. Moreover, it has been reported that the ability of low O_3_ concentrations to stimulate IL-2 secretion from T lymphocytes is related to their activation status [33].

### 3.4. The Modifications of Amino Acid, Carbohydrate, Lipid and Nucleotide Metabolites 

Metabolomic analysis provided original demonstrations of still unknown effects of low O_3_ concentrations on various amino acid, carbohydrate, lipid and nucleotide metabolites, whose modifications may be interpreted in the frame of the biological effects induced by O_3_ in blood. As discussed below, some of these metabolites underwent a quantitative decrease after O_3_ treatment, maybe due to their involvement as “sacrificial” molecules in the maintenance of the oxidant–antioxidant balance [5] or as substrates for energy production; on the other hand, other metabolites showed a significant increase after O_3_ treatment as factors required for the protective response against oxidative stress.

The modifications of the molecules following O_3_ exposure are mainly due to the oxidation of amino acids in the free amino group [38,39] and their quantitative changes in the plasma are often related to the role of these molecules in the antioxidant response triggered by the oxidative effect of O_3_. N-acetyl-L-alanine is a biologically available N-terminal capped form of L-alanine, an amino acid that exerts an antioxidant action by promoting the expression of the proteins heme oxygenase-1 (HO-1) and ferritin [40]; therefore, the increase in N-acetyl-L-alanine after O_3_ treatment could be due to its antioxidant role. Similarly, tryptophan metabolites play a role in the cellular redox response: 5-hydroxytryptophan was found to increase after O_3_ treatment, and it has been demonstrated that this compound acts as an antioxidant by transferring electrons to free radicals and directly scavenging H_2_O_2_, which is the precursor of −OH in the Fenton reaction system [41,42]. Tryptophan plays an important regulatory role in restoring the antioxidant system, enhancing the levels of GSH and glutathione peroxidase (GPx) in tissues [43], and the observed increase in picolinic acid, indoleacetic acid and indolelactic acid after O_3_ exposure is probably due to the enhanced catabolism of tryptophan [35]. The increase in ornithine and L-glutamic acid may contribute to the GSH synthesis, which is a key player in the cellular antioxidant response; its crucial role in the blood antioxidant response to O_3_ treatment is well-known [3,4,18] and an increase in GSH was reported after treatment with low O_3_ doses [44]. Modifications of the blood levels of L-threonine and 2-butenedioic acid (also known as maleic acid) have been reported under treatment with anti-inflammatory agents acting through the Nrf2/HO-1 signalling pathway [45]; on this basis, the increase in these metabolites found in the O_3_-treated samples could be related to the anti-inflammatory action of low O_3_ doses [5,46]. Similarly, S-carboxymethyl-L-cysteine (also known as carbocysteine) exhibits free-radical scavenging and anti-inflammatory properties [47,48] and shows an increase in ozonated blood. Finally, proline and its related metabolite, pyrrole-2-carboxylic acid, are involved in redox homeostasis, and the increased levels of these molecules after O_3_ treatment are likely linked to their protective effects against oxidative stress [49,50].

The modifications of molecules involved in carbohydrate metabolism are probably related to the stimulating effect of O_3_ on the glycolytic pathways. In fact, the D-glucose decrease agrees with the O_3_ ability to transiently increase the glycolysis rate with a consequent increase in the intracellular adenosine triphosphate (ATP) in erythrocytes [51]. Accordingly, also the glucose derivative glucuronolactone decreases in ozonated blood. Deoxypentitol and arabinofuranose are sugar-derived plant compounds, and their decrease may again be related to their use as energetic substrates. In this way, the reduction in pentanedioic acid (also known as glutaric acid) and tyrosine could be due to their consumption to supply the tricarboxylic acid cycle. The increase in malic acid, an intermediate of the Krebs cycle, may be due to the increased energy metabolism; in addition, malic acid is able to exert an antioxidant action by inhibiting the superoxide anion and downregulating the tumour necrosis factor α [52,53]. Aceturic acid (also known as N-acetyl glycine) is known to play a role in glucose metabolism, with its increase in blood having a strong correlation with oxidative metabolism, GSH biosynthesis and monosaccharide metabolism [54]; this could explain its increase in our O_3_-treated samples. 

Lipid molecules as well can be used as energy sources in the ATP production process, so the reduction in some lipid metabolites observed after O_3_ treatment may be due to their use as energy substrates. For instance, behenic and stearic acid (dietary fatty acids), campesterol (a phytosterol with a chemical structure similar to cholesterol), benzenepropanoic acid, and 3,5-bis(1,1-dimethylethyl)-4-hydroxy-methyl ester (a carboxylic ester derivative of a fatty acid) undergo reductions in O_3_-treated blood. Moreover, it is known that plasmatic unsaturated fatty acids are optimal substrates for O_3_, generating lipid peroxides that act as messengers to activate the Nrf2 response [11]. In this context, we found an increase in 2-hydroxy-3-methylbutyric acid (also known as 3-hydroxyisovaleric acid), involved in lipid peroxidation and β-oxidation [55], and in pimelic acid, a product of partially β-oxidized dietary odd-chain fatty acids [56]. Moreover, increased fatty acid oxidation has been found to increase the serum levels of oleic acid and linoleic acid [57], thus providing an explanation also for the observed increase in 9(E),11(E)-conjugated linoleic acid, trimethylsilyl ester (a linoleic acid derivative) in the O_3_-treated samples. Many lipid metabolites are involved in the antioxidant and anti-inflammatory pathways. Erythrono-1,4-lactone is an erythronic-acid-derived compound that is known to increase under oxidation conditions [58], and we can hypothesise that its increase in the O_3_-treated samples is related to this mechanism. Similarly, the increase in 3-octenoic acid (also known as carylic acid) could be related to its anti-inflammatory properties [59]. Butanoic acid, 2,4-bis[(trimethylsilyl)oxy]-, trimethylsilyl ester [60] and N-isobutyrylglycine [61] have been found to be upregulated under oxidative stress conditions, similarly to what was observed in our O_3_-treated samples. A decrease in (8Z,11Z,14Z)-icosa-8,11,14-trienoate (also known as 8,11,14-eicosatrienoic acid) has already been reported under oxidant–antioxidant altered conditions [62,63], probably due to its antioxidant activity [64,65]. Similarly, 4-hydroxybenzeneacetic acid, a carboxylic acid (also known as mandelic acid) has been demonstrated to have antioxidant properties [66], and 9-octadecen-1-ol (also known as linolenyl alcohol), a long chain fatty primary alcohol, acts as an antibacterial [67] and anti-inflammation agent [68]: all these molecules decreased in ozonised blood. Arachidic acid (also known as eicosanoid acid) is a lipid mediator involved in haemodynamics and inflammation with both anti-inflammatory and protective properties [69,70] and lanopalmitic acid (also known as hydroxyhexadecanoic acid) is involved in the Nrf2/HO-1 signalling pathway [45]: both of them have been found to decrease in O_3_-treated blood. Similarly, the tartaric acid reduction may be due to its utilisation as an antioxidant agent [71]. On the other hand, oleic acid, which has anti-inflammatory and antioxidant properties [72], and decanoic acid (also known as capric acid) able to reduce inflammatory cytokine production and oxidative stress [73] were found to increase in our O_3_-treated blood samples. Also, pentanoic acid (also known as valeric acid) that has antioxidant and anti-inflammatory properties [74] was found to increase in ozonated blood.

Concerning the nucleotide metabolites, our results showed a decrease in niacinamide (also known as nicotinamide or vitamin B3) and adenine, which are both precursors of nicotinamide adenine dinucleotide (NAD). More specifically, NAD may generate from two different pathways, one using tryptophan and the other using niacinamide [75]. NAD has the capability of heightening the production of nicotinamide adenine dinucleotide phosphate (NADPH), in order to have reducing equivalents to enhance the antioxidant capacity and increase the GSH levels [76]. The role of niacinamide in the blood antioxidant response has already been reported [3], and its decrease in ozonated blood could also be related to its antioxidant property [77]. Glyceric acid, which we found to increase in the O_3_-treated samples, is a NAD/NADPH upregulator [78]. It has been demonstrated that 9H-purin-6-ol (also known as hypoxantine) tends to increase in hypoxic conditions [79]; since in our O_3_-treated samples hypoxantine decreased, we may assume that this effect could be due to the hyperoxygenation induced by O_3_. The uridine decrease may be related to its capability to stabilise energy metabolism as well as to its antioxidant capacity [80,81]. 2,4-pyridinedicarboxylic acid is known to repress the hypoxia-inducible factor 1 (HIF-1) under normoxic condition, and ROS stimulate HIF-1 stabilisation [82]; it is therefore likely that the O_3_-derived oxidative stress induces an increase in 2,4-pyridinedicarboxylic acid.

## 4. Materials and Methods

### 4.1. Blood Ozonation

Due to the high amount of blood required for each experimental treatment (100 mL/sample), commercially available blood was used for this study. Sterile sheep blood was purchased from Microbiol Snc (Uta, CA, Italy). Coagulation was prevented via mechanical defibrination and the blood was used within 24 h from the collection. Before experimentation, the blood was heated to a temperature of 37 °C in a sterile incubator and then submitted to gaseous treatments. Aliquots of 100 mL were exposed to O_2_–O_3_ gas mixtures produced from medical-grade O_2_ by using an ECO3 apparatus (Ozoline, Brandizzo, TO, Italy). This apparatus operates at room temperature and allows photometric real-time control of the O_3_ concentration. The apparatus is connected to a system for blood treatment (Tecnoline, Concordia sulla Secchia, MO, Italy), which is composed of (i) a sterile and apyrogenic circuit made of tubes for blood draw, (ii) hollow fibres for continuous mixing of blood with gas, and (iii) a bag for collection of the ozonised blood. The whole system is internally coated with phosphorylcholine to minimise the interaction of blood with plastics. Through a peristaltic pump, the blood was ozonised at a constant flow of 20 mL/minute and then, reversing the rotation direction, recovered from the bag. The whole procedure required 20 min. O_3_ was used at the concentrations of 5 µg O_3_ and 10 µg O_3_/mL O_2_ (for a total of 50 µg O_3_ and 100 µg O_3_ administered to each blood sample, respectively). Pure O_2_ was administered to discern the effect induced by O_3_ from the O_2_-induced one. After gas treatment, the blood samples were submitted to different analyses (see below). The control samples did not undergo any treatment and were maintained at room temperature for the same time (20 min) required for the treatment with the O_3_ generator. All experiments were conducted in triplicate.

### 4.2. Haemogas Analysis

After gas treatment, 1 mL of blood was collected from each sample and then analysed with an Edan i15 Vet blood gas analyser (Scil Animal Care Company S.r.l., Treviglio, BG, Italy) following the manufacturer’s instructions to obtain the pO_2_, pCO_2_ and pH values. Each sample was analysed in triplicate.

### 4.3. Haemolysis Assay

To evaluate the haemolysis in the blood samples, the haemoglobin concentration was evaluated using a haemoglobin colorimetric assay kit (ab234046, Abcam, MA, USA) at the end of the gas treatment. Briefly, 20 µL of blood and plasma were incubated with 180 µL of haemoglobin detector buffer at room temperature for 15 min in 96-well plates. The absorbance was detected at 570 nm using a Stat Fax^®^ 4300 ChroMate^®^ (Awareness Technology, Inc., Palm City, FL, USA) and the haemolysis was expressed as the percentage of plasma haemoglobin over the total blood haemoglobin. Each sample was analysed in triplicate.

### 4.4. Total Antioxidant Capacity Assay

The antioxidant capacity of the blood samples was measured using a Total Antioxidant Capacity Assay kit (ab65329, Abcam, MA, USA) at the end of the gas treatment. Briefly, blood was diluted 1:2500 in bidistilled water and incubated according to the manufacturer’s instructions in 96-well plates. The absorbance was detected at 570 nm using a Stat Fax^®^ 4300 ChroMate^®^ (Awareness Technology, Inc., Palm City, FL, USA). The total antioxidant capacity was then calculated based on the absorbance of the samples with the standard curve. Each sample was analysed in triplicate.

### 4.5. Light Microscopy Analysis 

Morphological analysis of the erythrocytes was performed on blood smears by means of brightfield microscopy. Ten µL of control and treated blood samples were smeared on a slide and air-dried under a sterile hood. The smears were then stained with the May–Grunwald–Giemsa technique and observed with an Olympus BX51 microscope (Olympus Italia S.r.l., Segrate, MI, Italy) equipped with a QICAM Fast 1394 Digital 116 Camera (QImaging, Surrey, BC, Canada) for image acquisition. Each sample was analysed in triplicate.

### 4.6. Interleukin Assessment

The amounts of IL-2 and IL-6 were evaluated in the defibrinated plasma of the control and treated blood samples after blood cell sedimentation. The samples were maintained in a sterile incubator at 37 °C in a 5% CO_2_ humidified atmosphere for 2 h; then, 200 μL aliquots of plasma were stored at −80 °C until analysis. Interleukin quantitation was conducted via enzyme-linked immunosorbent assay (ELISA) on a Victor 3V mod. 1420 plate reader (Perkin Elmer, Waltham, MA, USA). Briefly, 200 μL aliquots of plasma were put onto a 96-well plate and the FineTest ELISA kits ESH0013 and ESH0019 (FineTest Biotech Inc., Boulder, CO, USA) were used as per the manufacturer’s recommendations to detect the sheep IL-2 and IL-6, respectively. The absorbance was read at 450 nm and the concentration of the target antigen in the sample was quantified. Samples were run in duplicate.

### 4.7. Metabolomics

Metabolomic analyses were performed on plasma aliquots obtained as described in Section 4.6. The plasma samples were prepared as previously reported [83]. Briefly, plasma metabolites were obtained via protein precipitation with cold acetonitrile/isopropanol/water, followed by derivatisation with methoxamine and BSTFA. Small molecules were analysed using a LECO Pegasus BT 4D GCXGC/TOFMS instrument (Leco Corp., St. Josef, MI, USA) equipped with a LECO dual stage quad jet thermal modulator. The GC part of the instrument was an Agilent 7890 gas chromatograph (Agilent Technologies, Palo Alto, CA, USA) equipped with a split/splitless injector. The first dimension column was a 30 m Rxi-5Sil (Restek Corp., Bellefonte, PA, USA) MS capillary column with an internal diameter of 0.25 mm and a stationary phase film thickness of 0.25 μm, and the second dimension chromatographic column was a 2 m Rxi-17Sil MS (Restek Corp., Bellefonte, PA, USA) with a diameter of 0.25 mm and a film thickness of 0.25 μm. High-purity helium (99.9999%) was used as the carrier gas, with a flow rate of 1.4 mL/minute. One μL of sample was injected in splitless mode at 250 °C. The temperature programme was as follow: the initial temperature was 100 °C for 2 min, then ramped 20 °C/minute up to 330 °C and then held at this value for 2 min. The secondary column was maintained at +5 °C relative to the GC oven temperature of the first column. The programming rate was the same for the two columns. Electron impact ionisation was applied (70 eV). The ion source temperature was set at 250 °C, the mass range was 25 to 550 *m*/*z* with an extraction frequency of 32 kHz. The acquisition rates were 200 spectra/s. The modulation period for the bi-dimensional analysis was 4 s for the entire run. The modulator temperature offset was set at +15 °C relative to the secondary oven temperature, while the transfer line was set at 280 °C. 

The chromatograms were acquired in total ion current mode. Peaks with a signal-to-noise (S/N) value lower than 500.0 were rejected. ChromaTOF version 5.31 was used for the raw data processing. Mass spectral assignment was performed by matching with the NIST MS Search 2.3 libraries and the FiehnLib. An in-house library of standards was also used for the small molecule identification. 

### 4.8. Statistical Analysis

Data for each variable were presented as the mean ± standard error (SE). The Mann–Whitney test was used for the statistical comparison between the gas-treated samples and controls as well as between the O_3_-treated samples and O_2_-treated ones. Statistical significance was set at *p* ≤ 0.05. MetaboAnalyst 5.0 software (www.metaboanalyst.org accessed on 5 December 2023) was used for the statistical analysis of the metabolomics data.

## 5. Conclusions

Our basic study on the molecular modifications induced in blood ex vivo by low O_3_ concentrations provides original information on the impact of mild ozonation on multiple metabolic pathways. The modified metabolites are mostly involved in the preservation of the oxidant–antioxidant balance and in energy production, according to the well-known effects of low O_3_ doses as enhancers of both the antioxidant/anti-inflammatory response and metabolic activity [5,19]. 

Remarkably, our findings demonstrate the ability of 5 µg O_3_/mL O_2_ to induce modifications in pO_2_, pCO_2_, pH, total antioxidant capacity, and many metabolites similarly to 10 µg O_3_/mL O_2_. The larger number of metabolites affected by 10 µg O_3_ in comparison to 5 µg O_3_ obviously suggests the stronger action of the higher O_3_ concentration; however, the effects of 5 µg O_3_ cannot be considered negligible and deserve attention, opening interesting perspectives to investigate, also in vivo, the therapeutic potential of very low O_3_ concentrations presently considered unsuitable because of the neutralising antioxidant potential of blood [4]. Blood treatment with reduced O_3_ concentrations would represent a further advancement of the clinical application of medical O_3_ in line with the low-dose O_3_ concept [21]. Moreover, the use of reduced O_3_ concentrations would be especially beneficial for patients with a chronically high level of oxidative stress: in these patients, mild O_3_ treatment would allow the restoration of a correct oxidant–antioxidant balance, avoiding the negative effect of high O_3_ concentrations on their blood antioxidant capacity.

## Figures and Tables

**Table 1:** Quantitative changes (decrease/increase: ↓/↑) in the metabolites of interest, as a consequence of the biological effects of O_3_.

5 μg O_3_	10 μg O_3_	5 μg O_3_ and 10 μg O_3_
(8Z,11Z,14Z)-Icosa-8,11,14-trienoate (↓)	1-Deoxypentitol (↓)	2-Butenedioic acid (↑)
Aceturic acid (↑)	2,4-Pyridinedicarboxylic acid (↑)	5-Hydroxytryptophan (↑)
D-Glucose (↓)	2-Hydroxy-3-methylbutyric acid (↑)	9(E),11(E)-Conjugated linoleic acid, trimethylsilyl ester (↑)
Erythrono-1,4-lactone (↑)	3-Octenoic acid (↑)	Adenine (↓)
	4-Hydroxybenzeneacetic acid (↓)	Arabinofuranose, 1,2,3,5-tetrakis-O- (↓)
	9H-Purin-6-ol (↓)	Benzenepropanoic acid, 3,5-bis(1,1-dimethylethyl)-4-hydroxy-, methyl ester (↓)
	9-Octadecen-1-ol (↓)	L-Glutamic acid, bis(trimethylsilyl) ester (↑)
	Arachidic acid (↓)	Malic acid (↑)
	Behenic acid (↓)	Niacinamide (↓)
	Butanoic acid, 2,4-bis[(trimethylsilyl)oxy]-, trimethylsilyl ester (↑)	Ornithine (↑)
	Campesterol (↓)	Pentanedioic acid (↓)
	Decanoic acid (↑)	Picolinic acid (↑)
	Glucuronolactone, trisO-(trimethylsilyl)- (↓)	Pyrrole-2-carboxylic acid (↑)
	Glyceric acid (↑)	Stearic Acid (↓)
	3-Indoleacetic acid (↑)	Tartaric acid (↓)
	Indole-3-lactic acid (↑)	
	Lanopalmitic acid (↓)	
	L-Proline (↑)	
	L-Threonine (↑)	
	L-Tyrosine (↓)	
	N-Acetyl-L-alanine (↑)	
	N-Isobutyrylglycine (↑)	
	Oleic acid (↑)	
	Pentanoic acid (↑)	
	Pimelic acid (↑)	
	S-Carboxymethyl-L-cysteine (↑)	
	Uridine (↓)	

## Data Availability

The data presented in this study are available on request from the corresponding author.

## Supplementary Materials

The supporting information can be downloaded at https://www.mdpi.com/article/10.3390/ijms242417175/s1.

## References

[1] Viebahn-Hansler R. (2007). The Use of Ozone in Medicine.

[2] Wolff H.H. (1974). Die Behandlung peripherer Durchblutungsstörungen mit Ozon. Erfahr. Hk..

[3] Bocci V., Zanardi I., Travagli V. (2011). Ozone: A new therapeutic agent in vascular diseases. Am. J. Cardiovasc. Drugs.

[4] Bocci V.A., Zanardi I., Travagli V. (2011). Ozone acting on human blood yields a hormetic dose-response relationship. J. Transl. Med..

[5] Sagai M., Bocci V. (2011). Mechanisms of Action Involved in Ozone Therapy: Is healing induced via a mild oxidative stress?. Med. Gas Res..

[6] Bocci V., Borrelli E., Travagli V., Zanardi I. (2009). The ozone paradox: Ozone is a strong oxidant as well as a medical drug. Med. Res. Rev..

[7] Guichardant M., Bacot S., Molière P., Lagarde M. (2006). Hydroxy-alkenals from the peroxidation of n-3 and n-6 fatty acids and urinary metabolites. Prostaglandins Leukot. Essent. Fatty Acids.

[8] Poli G., Schaur R.J., Siems W.G., Leonarduzzi G. (2008). 4-Hydroxynonenal: A membrane lipid oxidation product of medicinal interest. Med. Res. Rev..

[9] Long E.K., Picklo M.J. (2010). Trans-4-hydroxy-2-hexenal, a product of n-3 fatty acid peroxidation: Make some room HNE. Free Radic. Biol. Med..

[10] Bocci V., Valacchi G., Corradeschi F., Aldinucci C., Silvestri S., Paccagnini E., Gerli R. (1998). Studies on the biological effects of ozone: 7. Generation of reactive oxygen species (ROS) after exposure of human blood to ozone. J. Biol. Regul. Homeost. Agents.

[11] Tricarico G., Travagli V. (2021). The Relationship between Ozone and Human Blood in the Course of a Well-Controlled, Mild, and Transitory Oxidative Eustress. Antioxidants.

[12] Cattel F., Giordano S., Bertiond C., Lupia T., Corcione S., Scaldaferri M., Angelone L., De Rosa F.G. (2021). Ozone therapy in COVID-19: A narrative review. Virus Res..

[13] Niño-Sandoval T.C., Rocha N.S., Sarinho F.W., Vasconcelos C.F.M., Vasconcelos A.F.M., Vasconcelos B.C. (2021). Effect of autohemotherapy in the treatment of viral infections—A systematic review. Public Health.

[14] Tahmasebi S., Qasim M.T., Krivenkova M.V., Zekiy A.O., Thangavelu L., Aravindhan S., Izadi M., Jadidi-Niaragh F., Ghaebi M., Aslani S. (2021). The effects of oxygen-ozone therapy on regulatory T-cell responses in multiple sclerosis patients. Cell Biol. Int..

[15] An J.X., Wu G.P., Niu K., Wei Y.P., Liu H., Gao X.Y., Wu J.P., Wang Y., Renz H., Williams J.P. (2022). Treatment of Femoral Head Osteonecrosis with Ozone Therapy: Pilot Trial of a New Therapeutic Approach. Pain Physician.

[16] Shen W., Liu N., Ji Z., Fang H., Liu F., Zhang W., Yu X., Wang M., Zhang J., Wang X. (2022). Combining Ozonated Autohemotherapy with Pharmacological Therapy for Comorbid Insomnia and Myofascial Pain Syndrome: A Prospective Randomized Controlled Study. Pain Res. Manag..

[17] Serra M.E.G., Baeza-Noci J., Abdala C.V.M., Luvisotto M.M., Bertol C.D., Anzolin A.P. (2023). Clinical effectiveness of medical ozone therapy in COVID-19: The evidence and gaps map. Med. Gas. Res..

[18] Bocci V., Valacchi G. (2015). Nrf2 activation as target to implement therapeutic treatments. Front. Chem..

[19] Bocci V. (1994). Autohaemotherapy after treatment of blood with ozone. A reappraisal. J. Int. Med. Res..

[20] Bocci V., Valacchi G., Corradeschi F., Fanetti G. (1998). Studies on the biological effects of ozone: 8. Effects on the total antioxidant status and on interleukin-8 production. Mediators Inflamm..

[21] Viebahn-Hänsler R., Fernández O.S.L., Fahmy Z. (2012). Ozone in medicine: The low-dose ozone concept—Guidelines and treatment strategies. Ozone Sci. Eng..

[22] Viebahn-Haensler R., Fernández O.L. (2021). Ozone in medicine. The low-dose ozone concept and its basic biochemical mechanisms of action in chronic inflammatory diseases. Int. J. Mol. Sci..

[23] Travagli V., Zanardi I., Bocci V. (2006). A realistic evaluation of the action of ozone on whole human blood. Int. J. Biol. Macromol..

[24] Travagli V., Zanardi I., Silvietti A., Bocci V. (2007). A physicochemical investigation on the effects of ozone on blood. Int. J. Biol. Macromol..

[25] Skorup P., Fransson A., Gustavsson J., Sjöholm J., Rundgren H., Özenci V., Wong A.Y.W., Karlsson T., Svensén C., Günther M. (2022). Evaluation of an extracorporeal ozone-based bactericide system for the treatment of Escherichia coli sepsis. Intensive Care Med. Exp..

[26] Bocci V., Aldinucci C. (2006). Biochemical modifications induced in human blood by oxygenation-ozonation. J. Biochem. Mol. Toxicol..

[27] Pecorelli A., Bocci V., Acquaviva A., Belmonte G., Gardi C., Virgili F., Ciccoli L., Valacchi G. (2013). NRF2 activation is involved in ozonated human serum upregulation of HO-1 in endothelial cells. Toxicol. Appl. Pharmacol..

[28] Re L., Martínez-Sánchez G., Bordicchia M., Malcangi G., Pocognoli A., Morales-Segura M.A., Rothchild J., Rojas A. (2014). Is ozone pre-conditioning effect linked to Nrf2/EpRE activation pathway in vivo? A preliminary result. Eur. J. Pharmacol..

[29] Delgado-Roche L., Riera-Romo M., Mesta F., Hernández-Matos Y., Barrios J.M., Martínez-Sánchez G., Al-Dalaien S.M. (2017). Medical ozone promotes Nrf2 phosphorylation reducing oxidative stress and pro-inflammatory cytokines in multiple sclerosis patients. Eur. J. Pharmacol..

[30] Galiè M., Costanzo M., Nodari A., Boschi F., Calderan L., Mannucci S., Covi V., Tabaracci G., Malatesta M. (2018). Mild ozonisation activates antioxidant cell response by the Keap1/Nrf2 dependent pathway. Free Radic. Biol. Med..

[31] Cisterna B., Costanzo M., Nodari A., Galiè M., Zanzoni S., Bernardi P., Covi V., Tabaracci G., Malatesta M. (2020). Ozone Activates the Nrf2 Pathway and Improves Preservation of Explanted Adipose Tissue In Vitro. Antioxidants.

[32] Cisterna B., Costanzo M., Lacavalla M.A., Galiè M., Angelini O., Tabaracci G., Malatesta M. (2021). Low Ozone Concentrations Differentially Affect the Structural and Functional Features of Non-Activated and Activated Fibroblasts In Vitro. Int. J. Mol. Sci..

[33] Cappellozza E., Costanzo M., Calderan L., Galiè M., Angelini O., Tabaracci G., Malatesta M. (2021). Low ozone concentrations affect the structural and functional features of Jurkat T cells. Processes.

[34] Lacavalla M.A., Inguscio C.R., Cisterna B., Bernardi P., Costanzo M., Galiè M., Scambi I., Angelini O., Tabaracci G., Malatesta M. (2022). Ozone at low concentration modulates microglial activity in vitro: A multimodal microscopy and biomolecular study. Microsc. Res. Tech..

[35] Ding S., Duanmu X., Xu L., Zhu L., Wu Z. (2023). Ozone pretreatment alleviates ischemiareperfusion injury-induced myocardial ferroptosis by activating the Nrf2/Slc7a11/Gpx4 axis. Biomed. Pharmacother..

[36] Inguscio C.R., Cisterna B., Lacavalla M.A., Donati F., Angelini O., Tabaracci G., Malatesta M. (2023). Ozone and procaine increase secretion of platelet-derived factors in platelet-rich plasma. Eur. J. Histochem..

[37] Larini A., Bocci V. (2005). Effects of ozone on isolated peripheral blood mononuclear cells. Toxicol. In Vitro.

[38] Cataldo F. (2006). Ozone Degradation of Biological Macromolecules: Proteins, Hemoglobin, RNA, and DNA. Ozone Sci. Eng..

[39] Sharma V.K., Graham N.J.D. (2010). Oxidation of Amino Acids, Peptides and Proteins by Ozone: A Review. Ozone Sci. Eng..

[40] Grosser N., Oberle S., Berndt G., Erdmann K., Hemmerle A., Schröder H. (2004). Antioxidant action of L-alanine: Heme oxygenase-1 and ferritin as possible mediators. Biochem. Biophys. Res. Commun..

[41] Reyes-Gonzales M.C., Fuentes-Broto L., Martínez-Ballarín E., Miana-Mena F.J., Berzosa C., García-Gil F.A., Aranda M., García J.J. (2009). Effects of tryptophan and 5-hydroxytryptophan on the hepatic cell membrane rigidity due to oxidative stress. J. Membr. Biol..

[42] Keithahn C., Lerchl A. (2005). 5-hydroxytryptophan is a more potent in vitro hydroxyl radical scavenger than melatonin or vitamin C. J. Pineal Res..

[43] Xu K., Liu G., Fu C. (2018). The Tryptophan Pathway Targeting Antioxidant Capacity in the Placenta. Oxid. Med. Cell Longev..

[44] Buyuklu M., Kandemir F.M., Set T., Bakırcı E.M., Degirmenci H., Hamur H., Topal E., Kucukler S., Turkmen K. (2017). Beneficial Effects of Ozone Therapy on Oxidative Stress, Cardiac Functions and Clinical Findings in Patients with Heart Failure Reduced Ejection Fraction. Cardiovasc. Toxicol..

[45] Yuan A., Gong L., Luo L., Dang J., Gong X., Zhao M., Li Y., Li Y., Peng C. (2017). Revealing anti-inflammation mechanism of water-extract and oil of forsythiae fructus on carrageenan-Induced edema rats by serum metabolomics. Biomed. Pharmacother..

[46] Galiè M., Covi V., Tabaracci G., Malatesta M. (2019). The Role of Nrf2 in the Antioxidant Cellular Response to Medical Ozone Exposure. Int. J. Mol. Sci..

[47] Nogawa H., Ishibashi Y., Ogawa A., Masuda K., Tsubuki T., Kameda T., Matsuzawa S. (2009). Carbocisteine can scavenge reactive oxygen species in vitro. Respirology.

[48] Wang W., Zheng J.P., Zhu S.X., Guan W.J., Chen M., Zhong N.S. (2015). Carbocisteine attenuates hydrogen peroxide-induced inflammatory injury in A549 cells via NF-κB and ERK1/2 MAPK pathways. Int. Immunopharmacol..

[49] Krishnan N., Dickman M.B., Becker D.F. (2008). Proline modulates the intracellular redox environment and protects mammalian cells against oxidative stress. Free Radic. Biol. Med..

[50] Vettore L.A., Westbrook R.L., Tennant D.A. (2021). Proline metabolism and redox; maintaining a balance in health and disease. Amino Acids.

[51] Bocci V., Zanardi I., Huijberts M.S., Travagli V. (2011). Diabetes and chronic oxidative stress. A perspective based on the possible usefulness of ozone therapy. Diabetes Metab. Syndr..

[52] Tang X., Liu J., Dong W., Li P., Li L., Lin C., Zheng Y., Hou J., Li D. (2013). The cardioprotective effects of citric Acid and L-malic Acid on myocardial ischemia/reperfusion injury. Evid. Based Complement. Alternat. Med..

[53] Koriem K.M.M., Tharwat H.A.K. (2023). Malic Acid Improves Behavioral, Biochemical, and Molecular Disturbances in the Hypothalamus of Stressed Rats. J. Integr. Neurosci..

[54] Jonsson P., Antti H., Späth F., Melin B., Björkblom B. (2020). Identification of Pre-Diagnostic Metabolic Patterns for Glioma Using Subset Analysis of Matched Repeated Time Points. Cancers.

[55] Zhang Z., Yang M., Yin A., Chen M., Tan N., Wang M., Zhang Y., Ye H., Zhang X., Zhou W. (2020). Serum metabolomics reveals the effect of electroacupuncture on urinary leakage in women with stress urinary incontinence. J. Pharm. Biomed. Anal..

[56] Bennett M.J., Ragni M.C., Hood I., Hale D.E. (1992). Azelaic and pimelic acids: Metabolic intermediates or artefacts?. J. Inherit. Metab. Dis..

[57] Fatima T., Hashmi S., Iqbal A., Siddiqui A.J., Sami S.A., Basir N., Bokhari S.S., Sharif H., Musharraf S.G. (2019). Untargeted metabolomic analysis of coronary artery disease patients with diastolic dysfunction show disturbed oxidative pathway. Metabolomics.

[58] Björkblom B., Wibom C., Jonsson P., Mörén L., Andersson U., Johannesen T.B., Langseth H., Antti H., Melin B. (2016). Metabolomic screening of pre-diagnostic serum samples identifies association between α- and γ-tocopherols and glioblastoma risk. Oncotarget.

[59] Zhang X.S., Zhang P., Liu Y.H., Xu Q., Zhang Y., Li H.Z., Liu L., Liu Y.M., Yang X.Y., Xue C.Y. (2022). Caprylic Acid Improves Lipid Metabolism, Suppresses the Inflammatory Response and Activates the ABCA1/p-JAK2/p-STAT3 Signaling Pathway in C57BL/6J Mice and RAW264.7 Cells. Biomed. Environ. Sci..

[60] Orešič M., Hyötyläinen T., Herukka S.K., Sysi-Aho M., Mattila I., Seppänan-Laakso T., Julkunen V., Gopalacharyulu P.V., Hallikainen M., Koikkalainen J. (2011). Metabolome in progression to Alzheimer’s disease. Transl. Psychiatry.

[61] Shao X., Wang K., Liu X., Gu C., Zhang P., Xie J., Liu W., Sun L., Chen T., Li Y. (2016). Screening and verifying endometrial carcinoma diagnostic biomarkers based on a urine metabolomic profiling study using UPLC-Q-TOF/MS. Clin. Chim. Acta.

[62] Levy E., Rizwan Y., Thibault L., Lepage G., Brunet S., Bouthillier L., Seidman E. (2000). Altered lipid profile, lipoprotein composition, and oxidant and antioxidant status in pediatric Crohn disease. Am. J. Clin. Nutr..

[63] Ragino Y.I., Shramko V.S., Stakhneva E.M., Chernyak E.I., Morozov S.V., Shakhtshneider E.V., Polonskaya Y.V., Shcherbakova L.V., Chernyavskiy A.M. (2020). Changes in the blood fatty-acid profile associated with oxidative-antioxidant disturbances in coronary atherosclerosis. J. Med. Biochem..

[64] Chen S.J., Chuang L.T., Chen S.N. (2015). Incorporation of eicosatrienoic acid exerts mild anti-inflammatory properties in murine RAW264.7 cells. Inflammation.

[65] Vilakazi H., Olasehinde T.A., Olaniran A.O. (2021). Chemical Characterization, Antiproliferative and Antioxidant Activities of Polyunsaturated Fatty Acid-Rich Extracts from *Chlorella* sp. S14. Molecules.

[66] Parcheta M., Świsłocka R., Świderski G., Matejczyk M., Lewandowski W. (2022). Spectroscopic Characterization and Antioxidant Properties of Mandelic Acid and Its Derivatives in a Theoretical and Experimental Approach. Materials.

[67] Crout R.J., Gilbertson J.R., Gilbertson J.D., Platt D., Langkamp H.H., Connamacher R.H. (1982). Effect of linolenyl alcohol on the in-vitro growth of the oral bacterium Streptococcus mutans. Arch. Oral Biol..

[68] Kobori M., Nakayama H., Fukushima K., Ohnishi-Kameyama M., Ono H., Fukushima T., Akimoto Y., Masumoto S., Yukizaki C., Hoshi Y. (2008). Bitter gourd suppresses lipopolysaccharide-induced inflammatory responses. J. Agric. Food Chem..

[69] Serhan C.N., Dalli J., Colas R.A., Winkler J.W., Chiang N. (2015). Protectins and maresins: New pro-resolving families of mediators in acute inflammation and resolution bioactive metabolome. Biochim. Biophys. Acta.

[70] Harayama T., Shimizu T. (2020). Roles of polyunsaturated fatty acids, from mediators to membranes. J. Lipid Res..

[71] Jantwal A., Durgapal S., Upadhyay J., Joshi T., Kumar A., Nabavi S.M., Sanches Silva A. (2022). Tartaric acid. Antioxidants Effects in Health—The Bright and the Dark Side.

[72] Santa-María C., López-Enríquez S., Montserrat-de la Paz S., Geniz I., Reyes-Quiroz M.E., Moreno M., Palomares F., Sobrino F., Alba G. (2023). Update on Anti-Inflammatory Molecular Mechanisms Induced by Oleic Acid. Nutrients.

[73] Lee S.I., Kang K.S. (2017). Function of capric acid in cyclophosphamide-induced intestinal inflammation, oxidative stress, and barrier function in pigs. Sci. Rep..

[74] Jayaraj R.L., Beiram R., Azimullah S., Mf N.M., Ojha S.K., Adem A., Jalal F.Y. (2020). Valeric Acid Protects Dopaminergic Neurons by Suppressing Oxidative Stress, Neuroinflammation and Modulating Autophagy Pathways. Int. J. Mol. Sci..

[75] Denu J.M. (2007). Vitamins and aging: Pathways to NAD+ synthesis. Cell.

[76] Xie N., Zhang L., Gao W., Huang C., Huber P.E., Zhou X., Li C., Shen G., Zou B. (2020). NAD+ metabolism: Pathophysiologic mechanisms and therapeutic potential. Signal Transduct. Target Ther..

[77] Kamat J.P., Devasagayam T.P. (1999). Nicotinamide (vitamin B3) as an effective antioxidant against oxidative damage in rat brain mitochondria. Redox Rep..

[78] Hirvonen O.P., Kyröläinen H., Lehti M., Kainulainen H. (2021). Randomized Trial: D-Glyceric Acid Activates Mitochondrial Metabolism in 50–60- Year-Old Healthy Humans. Front. Aging.

[79] Hira H.S., Samal P., Kaur A., Kapoor S. (2014). Plasma level of hypoxanthine/xanthine as markers of oxidative stress with different stages of obstructive sleep apnea syndrome. Ann. Saudi Med..

[80] Krylova I.B., Bulion V.V., Selina E.N., Mironova G.D., Sapronov N.S. (2012). Effect of uridine on energy metabolism, LPO, and antioxidant system in the myocardium under conditions of acute coronary insufficiency. Bull. Exp. Biol. Med..

[81] Khezri M.K., Turkkan A., Koc C., Salman B., Levent P., Cakir A., Kafa I.M., Cansev M., Bekar A. (2021). Anti-Apoptotic and Anti-Oxidant Effects of Systemic Uridine Treatment in an Experimental Model of Sciatic Nerve Injury. Turk. Neurosurg..

[82] Mishur R.J., Khan M., Munkácsy E., Sharma L., Bokov A., Beam H., Radetskaya O., Borror M., Lane R., Bai Y. (2016). Mitochondrial metabolites extend lifespan. Aging Cell.

[83] Barberis E., Amede E., Tavecchia M., Marengo E., Cittone M.G., Rizzi E., Pedrinelli A.R., Tonello S., Minisini R., Pirisi M. (2021). Understanding protection from SARS-CoV-2 using metabolomics. Sci. Rep..

