# Supplementary material for: Modifications of Blood Molecular Components after Treatment with Low Ozone Concentrations
Source: Int J Mol Sci. 2023 Dec 6;24(24):17175. doi: 10.3390/ijms242417175 (PMC10742958; doi:10.3390/ijms242417175)
Supplement: Supplementary file 1 [file ijms-24-17175-s001.zip › ijms-2718382-supplementary.pdf]

Table S1. Metabolites identified in blood.

Metabolites	p value			fold change		
	5µg O_3 vs CTR	10µg O_3 vs CRT	O_2 vs CTR	5µg O_3 vs CTR	10µg O_3 vs CRT	O_2 vs CTR
(1S,4aS,4bS,7S,8aS,10aS)-7-Isopropyl-1,4a-dimethyltetradecahydrophenanthrene	0.669	0.700	0.490	2.918	0.971	0.513
(2-Mercaptopropionylamino)acetic acid	0.003	0.686	0.493	1.766	0.976	1.156
(7a-Isopropenyl-4,5-dimethyloctahydroinden-4-yl)methanol	0.396	0.709	0.353	0.800	1.089	1.238
(8Z,11Z,14Z)-Icosa-8,11,14-trienoate, O-TMS	0.002	0.482	0.324	0.013	0.882	1.706
(Diethylamino)ethanol	0.201	0.223	0.507	3.855	1.469	0.580
(n)-Phenylpropanolamine, N-trimethylsilyl-, trimethylsilyl ether	0.682	0.003	0.663	0.752	0.064	0.849
(Z)-Docos-9-enenitrile	0.148	0.215	0.590	1.543	1.442	0.891
{4-Methoxy-2-[(trimethylsilyl)oxy]phenyl}{2-[(trimethylsilyl)oxy]phenyl}methanone	0.942	0.887	0.942	3.828	0.966	0.182
1-(2-Methoxy-1-methylethoxy)-2-propanol	0.048	0.027	0.027	0.725	0.746	1.373
1-(Cyclohexylmethyl)-4-piperidinylamine	0.266	0.761	0.795	0.366	0.653	0.298
1-(p-Tolyl)butan-1-one	0.875	0.148	0.135	0.689	0.081	13.358
1,1,3,3,5,5,7,7-Octamethyl-7-(2-methylpropoxy)tetrasiloxan-1-ol	0.927	0.329	0.926	0.273	0.934	1.157
1,1'-Bicyclohexyl, 2-methyl-, trans-	0.222	0.974	0.001	0.445	1.101	12.032
1,1'-Biphenyl, 2,2',5,5'-tetramethyl-	0.723	0.264	0.849	0.696	0.391	1.110
1,1'-Biphenyl, 3,4-diethyl-	0.182	0.558	0.545	2.957	1.474	0.596
1,2,3-Butanetriol	0.027	0.237	0.215	2.682	1.660	1.825
1,2-Benzenedicarboxylic acid, bis(2-methylpropyl) ester	0.408	0.718	0.174	0.793	0.932	1.424
1,2-Cyclohexanedicarboxylic acid, cyclohexylmethyl nonyl ester	0.253	0.117	0.829	0.821	0.299	0.977
1,2-Cyclohexanedicarboxylic acid, dinonyl ester	0.996	0.216	0.450	1.034	0.853	1.106
1,2-Cyclopentanediol, 3-methyl-	0.764	0.973	0.696	0.690	1.236	1.646
1,2-Propanediol-1-phosphate,	0.557	0.006	0.370	2.263	0.030	0.758
1,3-Benzenediol, 4-ethyl-	0.428	0.028	0.186	0.599	0.142	2.345
1,3-Butanediol	0.298	0.695	0.567	1.807	0.771	0.474

1,3-Cyclohexanediol, cis-	0.369	0.312	0.153	0.672	0.474	2.561
1,3-Dibutyl-1,1,3,3-tetramethyldisiloxane	0.261	0.829	0.087	0.881	1.011	1.177
1,3-di-iso-propylnaphthalene	0.864	0.970	0.937	0.974	1.048	0.971
1,3-Propanediol	0.502	0.184	0.531	0.197	3.804	3.591
1,4,4,7a-Tetramethyl-2,4,5,6,7,7a-hexahydro-1H-indene-1,7-diol	0.479	0.517	0.855	1.741	1.951	1.051
1,4-Benzenedicarboxylic acid, bis(2-ethylhexyl) ester	0.718	0.950	0.574	1.035	1.013	1.088
1,4-Butanediol	0.878	0.954	0.586	1.404	0.998	0.908
1,4-Dioxaspiro[4.5]decane, 2-(trimethylsilyloxymethyl)-	0.266	0.007	0.093	0.207	0.028	10.028
1,5-Anhydroglucitol	0.579	0.605	0.575	1.102	0.922	1.084
1,6-Di(trimethylsilyl)hexane	0.318	0.452	0.306	0.596	1.124	1.740
1,6-Dioxacyclododecane-7,12-dione	0.245	0.189	0.272	0.796	0.779	1.205
10,18-Bisnorabieta-5,7,9(10),11,13-pentaene	0.864	0.392	0.222	1.021	2.371	2.870
10,18-Bisnorabieta-8,11,13-triene	0.369	0.951	0.061	0.753	1.980	2.387
10-Nonadecenoic acid, (Z)-	0.093	0.712	0.059	1.289	1.055	1.389
11,14-Eicosadienoic acid	0.467	0.573	0.740	1.820	1.583	1.647
11-Eicosenoic acid, (E)-	0.106	0.790	0.917	2.828	1.570	1.051
12-Hydroxyoctadecanoic acid	0.708	0.052	0.994	1.075	0.630	1.015
13-Docosenamide, (Z)-	0.412	0.344	0.196	0.876	0.821	1.291
13-Keto-9Z,11E-octadecadienoic acid	0.205	0.106	0.086	1.247	2.474	1.308
16,17-Dinorpisferal A	0.343	0.838	0.359	0.580	1.118	1.677
17a-Hydroxyprogesterone	0.903	0.751	0.849	0.575	1.100	1.933
18-Norabietane	0.15	0.019	0.463	4.248	4.747	0.453
1-Butanamine, N,N-diethyl-	0.313	0.249	0.178	0.873	0.863	1.179
1-cis-Monovaccenylglycerol bis-trimethylsilyl ester	0.171	0.072	0.753	1.848	5.083	1.024
1-Deoxypentitol	0.076	0.008	0.122	0.728	0.499	1.230
1-Dimethylaminohexane	0.461	0.832	0.088	0.910	0.976	1.244
1-Dimethylthexylsilyloxydecane	0.842	0.600	0.634	0.827	1.580	0.754
1-Docosene	0.432	0.499	0.040	1.875	2.182	0.026
1-Dodecanamine, N,N-dimethyl-	0.628	0.240	0.735	0.923	0.800	1.055
1-Eicosanol	0.305	0.703	0.464	0.332	4.495	0.376

1-Ethyl-1-tetradecyloxy-1-silacyclopentane	0.445	0.078	0.051	2.959	0.137	0.260
1-Heptanamine	0.379	0.307	0.614	1.316	1.435	0.801
1-Hexacosene	0.763	0.564	0.234	1.259	0.980	1.966
1-Hexadecanol	0.201	0.296	0.392	1.837	1.566	0.751
1-Hexadecylamine	0.91	0.604	0.909	0.429	0.818	0.902
1H-Indene, 1-ethylidene-	0.195	0.157	0.025	0.835	0.865	1.319
1H-Indole-3-carboxamide, 1-(5-fluoropentyl)-N-(phenylmethyl)-	0.749	0.345	0.434	1.485	0.588	0.750
1H-Indole-3-carboxylic acid, 1-pentyl-, 8-quinolinyl ester	0.949	0.942	0.787	0.519	0.601	0.559
1H-Naphtho[2,1-b]pyran, 3-ethenyldodecahydro-3,4a,7,7,10a-pentamethyl-, [3R-(3a,4aa,6aa,10aa,10ba)]-	0.305	0.372	0.498	0.700	1.184	1.192
1-Iodo-2-methylundecane	0.385	0.992	0.180	0.608	0.937	3.033
1-Methyl-L-histidine	0.461	0.001	0.296	1.349	0.180	1.214
1-Methyl-N,N-bis(trimethylsilyl)-4-[(trimethylsilyl)oxy]-1H-imidazol-2-amine	0.891	0.389	0.146	0.981	0.896	1.225
1-Monooleoylglycerol	0.270	0.744	0.493	2.743	5.098	1.656
1-Monopalmitin	0.106	0.204	0.049	0.830	3.915	1.288
1-Monopalmitylglycerol bis-trimethylsilyl ether	4.69E-05	0.004	0.224	4.654	14.056	0.285
1-Nonene, 4,6,8-trimethyl-	0.911	0.434	0.286	1.057	0.812	1.427
1-Octadecanol	0.820	0.703	0.269	0.936	0.908	1.292
1-Octanol	0.694	0.450	0.653	1.479	1.059	2.984
1-Octanol, 2-butyl-	0.891	0.999	0.771	1.011	1.011	0.923
1-Octanol	0.730	0.318	0.371	1.044	0.870	1.162
1-Pentadecene	0.585	0.413	0.308	2.171	1.259	0.387
1-Pentanol, 2-ethyl-4-methyl-	0.339	0.028	0.093	0.729	0.039	4.410
1-Pentene, 2,3-dimethyl-	0.222	0.420	0.196	0.380	2.314	2.763
1-Phenyl-1,2-ethanediol	0.620	0.868	0.427	0.873	1.001	1.224
1-Piperazineethanol, 4-dibenzo[b,f][1,4]thiazepin-11-yl-	0.592	0.269	0.742	1.637	0.377	0.784
1-Piperidin-1-ylpropan-2-ol, tert-butyldimethylsilyl ether	0.353	0.026	0.561	0.669	0.301	1.123
1-Piperidin-1-ylpropan-2-ol, trimethylsilyl ether	0.965	0.899	0.205	0.969	1.008	0.743

1-Tricosanol	0.189	0.611	0.678	1.464	0.932	0.930
1-Undecene, 5-methyl-	0.343	0.938	0.054	0.685	1.277	1.118
2-(2-Butoxyethoxy)acetic acid	0.255	0.765	0.457	1.834	0.827	0.405
2-(2-Trimethylsilyloxy-5-methylphenyl)benzotriazole	0.011	0.030	0.537	7.444	4.450	0.868
2(3H)-Furanone, 5-dodecyldihydro-	0.114	0.087	0.354	0.328	0.826	1.641
2(3H)-Furanone, dihydro-5,5-dimethyl-4-(3-oxobutyl)-	0.430	0.180	0.328	0.759	0.565	1.323
2-(3-Pyridyl)-4-methylthiazole-5-carboxylic acid	0.165	0.223	0.004	3.688	1.196	0.146
2-(Cholest-5-en-3-yloxy)ethanol	0.044	0.018	0.062	0.638	0.510	1.441
2,2'-Bipyridine	0.612	0.570	0.199	0.929	0.916	1.206
2',3,4,5,6'-Pentamethoxychalcone	0.416	0.321	0.072	0.980	2.894	5.138
2,3,4-Trihydroxybutyric acid tetrakis(trimethylsilyl) deriv., (, (R*,R*)-)	0.572	0.821	0.669	1.065	1.020	1.064
2,3-Dimethyl-3-pentanol	0.065	0.968	0.947	6.866	3.068	0.298
2,3-Dimethyl-5-oxohexanethioic acid, S-t-butyl ester	0.697	0.079	0.338	0.958	0.851	1.201
2,3'-Dipyridyl	0.220	0.148	0.235	0.787	0.730	1.857
2,3-Pentanedione	0.837	0.237	0.473	0.897	0.391	0.441
2,4(1H,3H)-Pyrimidinedione, 6-amino-1,3-di-2-propenyl-	0.003	0.002	0.289	0.040	0.037	0.694
2,4,4-Trimethyl-3-(3-methylbutyl)cyclohex-2-enone	0.436	0.386	0.081	0.907	0.943	1.229
2,4,6-Trimethoxybenzaldehyde	0.082	0.207	0.191	0.281	0.528	2.192
2,4,6-Tris(cyclohexyl)hept-1-ene	0.412	0.004	0.086	1.802	12.744	1.264
2,4,7,9-Tetramethyl-5-decyne-4,7-diol	0.290	0.312	0.348	1.880	1.686	0.645
2,4'-Bipyridine	0.410	0.436	0.375	1.315	1.291	0.684
2,4-Dibenzoylrezorcinol, bis(trimethylsilyl) ether	0.817	0.089	0.529	1.089	0.783	0.570
2,4-Dimethoxybenzylamine	0.237	0.813	0.062	0.527	0.974	3.435
2,4-Di-tert-butylphenoxytrimethylsilane	0.430	0.028	0.125	0.888	0.764	1.195
2,4-Pyridinedicarboxylic acid	0.133	0.013	0.135	5.143	7.326	0.320
2,4-Thiazolidinedione	0.205	0.432	0.219	1.430	1.926	0.230
2,5-Bis((trimethylsilyl)oxy)pyrazine	0.004	0.001	0.054	0.093	0.078	1.391
2,5-Cyclohexadiene-1,4-dione, 2,6-bis(1,1-dimethylethyl)-	0.219	0.120	0.395	1.325	1.413	0.865

2,5-Dimethoxy-4-isopropylthiophenethylamine	0.136	0.487	0.414	3.934	1.913	0.539
2,5-Dimethoxybenzoic acid	0.038	0.001	0.001	0.415	0.621	3.419
2,5-Dimethoxymandelic acid, di-TMS	0.912	0.169	0.207	2.947	11.872	2.382
2,5-Dimethoxyphenol, TMS	0.095	0.139	0.049	1.454	1.393	1.203
2,5-di-tert-Butyl-1,4-benzoquinone	0.420	0.101	0.300	0.739	0.550	1.447
2,5-Di-tert-butyl-4-((trimethylsilyl)oxy)phenol	0.073	0.034	0.015	0.767	0.636	1.400
2,5-Hexanediol	0.271	0.512	0.293	1.385	1.168	0.856
2,6,10-Trimethyltridecane	0.124	0.091	0.470	1.301	1.051	0.931
2,6,8-Trimethyl-4-nonyl acetate	0.992	0.691	0.496	1.212	1.300	0.248
2,6-Diisopropylnaphthalene	0.707	0.790	0.476	0.912	0.932	1.240
2,6-Dimethyldecane	0.513	0.455	0.920	0.839	0.828	0.994
2,6-Di-tert-butyl-4-hydroxy-4-methylcyclohexa-2,5-dien-1-one	0.116	0.137	0.903	2.347	1.689	1.252
2-[(1-Hexyl-1H-1,2,3-triazol-4-yl)methyl]-4-[methyl(propyl)amino]isothiazolidine 1,1-dioxide	0.070	0.086	0.018	0.647	1.799	1.796
2-[Methyl(4-piperidinyl)amino]ethanol	0.471	0.009	0.259	0.592	0.076	2.708
2-Allyl-1,4-dimethoxybenzene	0.281	0.199	0.087	0.805	0.733	1.390
2-Aminobutanoic acid	0.828	0.383	0.521	2.481	0.623	0.835
2-Bromo dodecane	0.592	0.878	0.037	0.912	1.270	1.430
2-Butanone, 3-methoxy-3-methyl-	0.956	0.323	0.863	1.216	0.341	1.242
2-Butenedioic acid, (Z)-	0.030	0.005	0.560	1.650	1.979	1.084
2-Chloro-9-[2-deoxy-3,5-bis-O-(trimethylsilyl)pentofuranosyl]-9H-purin-6-amine	0.872	0.201	0.005	0.990	0.635	2.013
2-Cyclohexen-1-one, 3,5-dimethyl-	0.247	0.044	0.116	0.857	0.767	1.221
2-Decanone	0.085	0.267	0.179	0.842	0.601	1.888
2-Decene, 8-methyl-, (Z)-	0.279	0.914	0.319	0.853	0.997	1.430
2'-Desoxyuridine	0.902	0.051	0.273	1.036	0.265	1.177
2-Dodecene, (E)-	0.347	0.369	0.073	0.438	0.419	6.173
2-Ethyl-1-dimethyl(chloromethyl)silyloxyhexane	0.221	0.184	0.041	0.451	0.310	8.053
2-Ethyl-1-hexanol	0.361	0.247	0.041	105.280	28.024	0.010
2-Ethyl-3-hydroxypropionic acid, di-TMS	0.849	0.706	0.097	1.024	0.944	1.264
2-Ethylacridine	0.305	0.323	0.008	0.332	0.341	0.052
2-Ethylhexanol	0.606	0.194	0.991	1.309	2.385	1.343

2-Heptadecanol, acetate	0.312	0.849	0.549	0.366	0.239	0.560
2-Hexenal	0.542	0.837	0.017	6.268	3.542	0.198
2-Hexenal, (E)-	0.954	0.109	0.304	0.975	2.379	1.964
2-Hydroxy-3-methylbutyric acid	0.082	0.032	0.179	1.352	3.521	1.894
2-Hydroxy-5-methoxybenzaldehyde	0.439	0.345	0.003	0.835	0.866	1.321
2-Hydroxybutyric acid	0.077	0.206	0.038	0.824	0.854	2.234
2-Hydroxyisocaproic acid	0.032	0.024	0.685	4.152	34.901	0.819
2-Hydroxymethyl-17a-methylandrosta-1,4-diene-11a,17a-diol-3-one	0.205	0.432	0.001	1.304	1.926	0.166
2-Isopropyl-5-methyl-1-heptanol	0.114	0.725	0.172	0.112	1.412	56.263
2-Ketobutyric acid eo-tms	0.292	0.308	0.174	0.665	0.888	1.769
2-Monostearin	0.228	0.004	0.528	0.289	0.016	1.422
2-Naphthol	0.585	0.303	0.788	0.928	0.922	1.028
2-n-Propyl-1-heptanol	0.155	0.627	0.213	0.472	1.243	2.171
2-Octanol	0.573	0.970	0.569	1.354	0.354	8.614
2-Octene, 4-ethyl-	0.007	0.118	0.378	0.465	0.758	1.457
2-Octenoic acid	0.557	0.149	0.455	0.749	2.215	0.798
2-Oleoylglycerol	0.113	0.396	0.359	0.159	2.449	3.384
2-Palmitoylglycerol	0.975	0.096	0.182	0.992	2.427	2.060
2-Pentamethyldisilanyloxybutane	0.128	0.111	0.573	2.226	2.534	1.510
2-Pentenedioic acid, 2-[(trimethylsilyl)oxy]-, bis(trimethylsilyl) ester	0.259	0.658	0.339	1.432	0.895	1.376
2-Propanamine, N-ethyl-	0.366	0.761	0.163	0.881	0.964	1.227
2-Propenoic acid, 2-[(trimethylsilyl)oxy]-, anhydride with bis(trimethylsilyl) hydrogen phosphate	0.986	0.245	0.013	0.892	0.444	0.143
2-Propenoic acid, 2-[(trimethylsilyl)oxy]-, trimethylsilyl ester	0.593	0.861	0.077	1.071	0.983	1.233
2-Propenoic acid, 2-methyl-, 3,3,5-trimethylcyclohexyl ester	0.076	0.003	0.011	0.746	0.668	1.480
2-Propyl-1-pentanol	0.468	0.318	0.233	0.775	1.668	1.905
2-Propyl-1-pentanol	0.967	0.998	0.514	1.052	1.030	1.319
2-Pyrrolidinone	0.215	0.246	0.094	1.184	1.294	1.388
2-Trimethylsiloxy-4-methyl-1,3-pentadiene	0.515	0.518	0.578	0.655	6.189	1.339
2-Undecanethiol, 2-methyl-	0.199	0.670	0.912	0.445	1.129	1.227

2-Undecene, (E)-	0.408	0.233	0.591	0.808	2.283	0.683
3-(2-Hydroxyethyl)imidazole-2-thione	0.006	0.152	0.453	0.849	0.600	1.128
3-(2-Methyl-1-trimethylsilylmethyl-2-propenyl)cyclohexanone	0.078	0.006	0.071	1.726	2.645	1.886
3-(3,4-Dimethylthieno[2,3-b]thiophen-2-yl)-1-methyl-5-methylsulfanyl-1H-pyrazole	0.883	0.008	0.319	2.484	0.077	0.625
3-(5-Oxooxolan-2-yl)propanoic acid	0.521	0.434	0.343	1.082	1.119	1.169
3-(5-tert-Butyl-3-isoxazolyl)urea	0.778	0.299	0.055	0.975	0.898	1.231
3(N,N-Dimethylmyristylammonio)propanesulfonate	0.956	0.487	0.967	0.709	0.408	0.911
3,2'-Dihydroxychalcone	0.690	0.308	0.168	1.119	0.523	2.437
3,4,5-Trihydroxypentanoic acid, tetrakis(trimethylsilyl)-	0.570	0.948	0.495	1.153	0.906	0.843
3,4-Dimethoxymandelic acid, di-TMS	0.493	0.002	0.173	0.903	0.493	1.348
3,4-dimethyl-5-(3,4-methylenedioxy)phenyloxazolidine	0.015	0.077	0.052	0.711	0.797	1.373
3,5,5-Trimethyl-1-hexanol	0.224	0.238	0.363	1.412	2.312	1.511
3,5-Dimethyl-2-phenylpyridine	0.191	0.198	0.212	1.194	1.276	1.186
3,5-di-tert-Butyl-4-hydroxyacetophenone	0.999	0.002	0.050	1.320	5.879	2.537
3,7-dioxa-2,8-disilanonane, 2,2,8,8-tetramethyl-5,5-bis[[(trimethylsilyl)oxy]methyl]-	0.955	0.044	0.556	1.124	1.133	1.048
3-Amino-5-morpholinomethyl-2-oxazolidinone	0.032	0.062	0.126	4.900	3.473	0.415
3-Aminobenzoic acid	0.703	0.431	0.027	0.955	0.864	1.507
3a-TRIMETHYLSILOXY-5a,6a-EPOXYCHOLESTANE	0.510	0.113	0.207	0.896	0.065	2.528
3-Dodecene, (E)-	0.995	0.584	0.721	1.235	0.046	1.338
3-Ethoxy-1,1,1,5,5,5-hexamethyl-3-(trimethylsiloxy)trisiloxane	0.678	0.733	0.839	2.115	0.968	0.530
3-Heptene, 2,2,3,5,6-pentamethyl-	0.245	0.283	0.555	0.739	0.782	0.867
3-Hydroxy-3-phenylpropanoic acid	0.075	0.020	0.447	0.686	0.619	1.117
3-Hydroxybutyric acid	0.687	0.385	0.131	1.079	1.124	1.203
3-Hydroxyflavone	0.103	0.004	0.321	10.071	0.064	0.710
3-Hydroxyisovaleric acid	0.087	0.037	0.721	1.432	1.566	0.949
3-Indoleacetic acid	0.083	0.001	0.480	1.695	3.148	0.900
3-Methyl-1-cyclohexanecarboxylic acid, trimethylsilyl ester (stereoisomer 2)	0.839	0.048	0.394	0.984	1.169	1.119

3-Methylbutanoic acid	0.767	0.074	0.394	3.302	11.399	0.333
3-Methylene-cyclopropane-1,2-carboxylic acid peroxide t-butyl ester	8.96E-05	0.118	0.189	4.475	2.980	0.331
3-Methylvaleric acid	0.432	0.564	0.492	1.296	1.105	0.839
3-Octenoic acid	0.807	0.026	0.616	1.080	1.558	1.063
3-Oxostearic acid	0.609	0.188	0.086	2.174	3.362	1.842
3-Phenyllactic acid	0.411	0.271	0.596	0.823	1.570	1.131
3-Undecene, 9-methyl-, (E)-	0.269	0.998	0.262	0.552	1.499	4.079
4-((Bis(trimethylsilyl)amino)methyl)-N-(trimethylsilyl)benzenesulfonamide	0.436	0.465	0.723	1.172	0.831	0.958
4-(Pentamethyldisilanyl)phenyltrimethylsilylmethanol	0.860	0.029	0.057	3.335	0.037	0.189
4,4'-Ethylenedi-m-toluidine	0.001	9.63E-05	0.001	0.119	0.122	6.893
4,6-Dioxa-5-aza-2,3,7,8-tetrasilanonane, 2,2,3,3,7,7,8,8-octamethyl-	0.974	0.315	0.400	0.991	0.287	0.760
4,8,12-Trimethyltridecan-4-olide	0.668	0.305	0.279	0.949	0.717	1.138
4-[2-(4,7-Dimethoxy-2H-1,3-benzodioxol-5-yl)ethenyl]-2-methoxyphenol	0.828	0.486	0.364	0.954	0.855	1.200
4-Amino-6-hydroxypyrimidine	0.192	0.599	0.343	1.842	1.234	1.978
4-Azido-2-nitrobutyric acid, 2,6-di-t-butyl-4-methoxyphenyl ester	0.608	0.487	0.383	1.148	0.869	1.420
4b,8-Dimethyl-2-isopropylphenanthrene, 4b,5,6,7,8,8a,9,10-octahydro-	0.425	0.965	0.285	0.720	1.155	1.630
4-Chloro-2,5-dimethoxyamphetamine	0.007	0.129	0.029	0.497	0.750	1.661
4-Chloro-3-n-hexyltetrahydropyran	0.992	0.567	0.773	0.846	1.153	0.980
4-Fluoroaniline, N-trimethylsilyl-	0.052	0.061	0.088	2.238	0.212	0.509
4'-Hydroxyacetophenone	0.470	0.003	0.003	0.826	0.024	1.562
4-Hydroxybenzaldehyde	0.512	0.497	0.157	1.139	1.139	1.335
4-Hydroxybenzeneacetic acid	0.376	0.032	0.674	0.844	0.723	1.059
4-Hydroxybutanoic acid	0.569	0.247	0.026	1.186	1.317	1.461
4'-Hydroxyflavanone	0.222	0.282	0.196	0.380	4.781	2.763
4-Hydroxyphenyllactic acid	0.233	0.107	0.283	0.764	0.683	1.195
4-Iodo-3-nitrobenzamide	0.673	0.041	0.239	0.480	0.016	0.670
4-Methyl-2-pentanol	0.618	0.025	0.879	2.120	6.980	0.676

4-Methylvaleric acid	0.401	0.069	0.282	0.823	0.664	1.315
4-Octene, 2,2,3,7-tetramethyl-, [S-(E)]-	0.981	0.344	0.863	0.473	0.341	1.511
4-Oxohexanoic acid, tert-butyldimethylsilyl ester	0.298	0.061	0.071	0.885	0.815	1.261
4-Phenanthrenol, 1,2,3,4-tetrahydro-4-methyl-	0.475	0.097	0.059	0.907	0.803	1.281
4-Pyridinecarbonitrile	0.894	0.091	0.001	1.120	0.374	0.344
4-Pyridinol	0.644	0.767	0.780	1.138	0.930	1.069
4-Pyrimidinamine, N-(trimethylsilyl)-2-[(trimethylsilyl)oxy]-	0.423	0.891	0.169	0.808	1.033	2.020
4-sec-Butyl-2,6-di-tert-butylphenol, trimethylsilyl ether	0.643	0.001	0.481	0.932	0.105	1.069
4-Trimethylsiloxy(trimethylsilyl)valerate	0.989	0.987	0.816	0.932	0.933	1.241
4-Undecene, 3-methyl-, (Z)-	0.087	0.523	0.256	0.145	1.325	2.202
5,7-Dimethoxy-3-hydroxyflavone	0.445	0.794	0.160	0.120	1.054	50.133
5,8,11-Eicosatriynoic acid, tert-butyldimethylsilyl ester	0.168	0.153	0.308	1.396	0.390	1.316
5a-Estran-3a-ol-17-one, di-trimethylsilyl	0.789	0.018	0.150	0.948	0.262	0.779
5-Amino-2-cyanobenzotrifluoride	0.951	0.290	0.405	1.001	0.802	1.112
5-Cholesten-3a, 7a-bis[(trimethylsilyl)oxy]-	0.334	0.001	0.749	0.959	0.368	0.959
5-Cycloocten-1-amine, 2-(3-nitro-2-pyridyloxy)-N-methyl--N-(3-nitro-2-pyridyl)-	0.033	0.774	0.233	2.059	1.003	0.770
5H-1-Pyrindine, 6,7-dihydro-	0.314	0.410	0.117	0.882	0.918	1.291
5-Hydroxyflavone	0.814	0.975	0.807	5.300	7.371	0.992
5-Hydroxyindoleacetic acid	0.853	0.157	0.081	0.885	0.431	3.018
5-Hydroxyquinoline, trimethylsilyl ether	0.008	0.117	0.637	21.76	3.318	0.503
5-Hydroxytryptophan	0.001	5.35E-05	0.086	4.711	4.832	1.308
5-Hydroxytryptophol	0.232	0.295	0.099	2.142	1.625	4.710
5-Iodopentan-2-one	0.126	0.023	0.078	0.815	0.773	1.206
5S,15S-Dihydroxy-6E,8Z,11Z,13E-eicosatetraenoic acid	0.518	0.695	0.196	2.960	10.804	0.010
5-Trimethylsilyloxy-n-valeric acid, trimethylsilyl ester	0.640	0.304	0.448	0.938	1.091	1.097
6H-Purin-6-one, 1,7-dihydro-	0.727	0.140	0.538	1.009	5.738	4.581
6-Hydroxyflavone	0.620	0.281	0.424	0.606	1.667	0.787
6-Hydroxyflavone-a-D-glucoside	0.106	0.721	3.34E-06	0.296	0.980	6.630

6-Oxoheptanoic acid	0.533	0.920	0.058	0.910	1.004	1.377
7,10,13,16-Docosatetraenoic acid, (Z)-	0.672	0.930	0.831	0.269	0.486	1.006
7,9-Di-tert-butyl-1-oxaspiro(4,5)deca-6,9-diene-2,8-dione	0.529	0.135	0.152	0.922	0.833	1.171
7-Acetyl-6-ethyl-1,1,4,4-tetramethyltetralin	0.360	0.937	0.147	0.737	1.003	1.596
7-Hydroxyisoflavone	0.627	0.243	0.877	1.299	3.429	1.400
7-Isopropyl-1,1,4a-trimethyl-1,2,3,4,4a,9,10,10a-octahydrophenanthrene	0.349	0.909	0.183	0.658	1.295	1.882
7-Methyl-Z-tetradecen-1-ol acetate	0.388	0.406	0.561	3.650	60.716	4.357
7-Oxooctanoic acid	0.963	0.426	0.086	1.005	0.763	1.297
8,8,9-Trimethyl-deca-3,5-diene-2,7-dione	0.536	0.257	0.132	0.925	0.904	1.186
8-Methylnonanoic acid, trimethylsilyl ester	0.071	0.016	0.227	1.407	1.483	1.186
9(E),11(E)-Conjugated linoleic acid, trimethylsilyl ester	0.004	0.006	0.295	2.069	1.861	1.152
9,12,15-Octadecatrien-1-ol, (Z,Z,Z)-	0.491	2.68E-05	0.086	1.875	5.163	1.474
9,12-Octadecadienoic acid (Z,Z)-	0.767	0.098	0.246	5.254	12.439	2.164
9-Hexadecenoic acid, (Z)-	0.738	0.090	0.072	2.020	0.184	0.390
9H-Fluorene, 9-methylene-	0.049	0.404	0.012	0.486	0.753	2.069
9H-Purin-6-ol	0.855	0.001	0.128	1.416	0.121	0.731
9-Octadecen-1-ol, (Z)-	0.765	0.013	0.495	1.059	0.041	1.182
9-Octadecenamide, (Z)-	0.820	0.407	0.213	1.028	1.254	1.272
9-Octadecenoic acid (Z)-, methyl ester	0.356	0.400	0.273	0.431	1.815	3.294
9-Octadecenoic acid, (E)-	0.323	0.763	0.353	0.588	0.896	1.577
a-Carboline-1-propionic acid	0.417	0.128	0.117	0.263	0.069	15.585
Acetamide, N,N-diethyl-	0.608	0.814	0.133	0.929	1.027	1.252
Acetamide, N-[4-(trimethylsilyl)phenyl]-	0.026	0.010	0.383	0.419	0.154	1.139
Acetamide	0.151	0.284	0.199	0.860	0.882	1.466
Acetoacetic acid	0.021	0.393	0.104	1.263	0.842	1.356
Acetophenone	0.514	0.185	0.143	0.924	0.865	1.186
Aceturic acid	0.034	0.566	0.296	3.674	0.892	1.602
a-D-(+)-Talopyranose	0.069	0.265	0.796	4.416	2.024	1.204
a-D-Allopyranose	0.158	0.943	0.976	7.052	0.655	1.063
Adenine	0.007	0.034	0.892	0.026	0.197	1.005

Adenine	0.056	0.001	0.245	0.300	0.009	1.066
a-D-Glactopyranose	0.433	0.191	0.086	1.802	4.345	1.542
a-D-Glucopyranoside, methyl 2-(acetylamino)-2-deoxy-3-O-(trimethylsilyl)-, cyclic methylboronate	0.809	0.329	0.310	1.343	0.377	2.870
Adrenaline	0.845	0.172	0.929	2.826	0.008	1.503
a-Hydroxyisobutyric acid	0.505	0.190	0.086	1.875	3.710	1.746
Allopseudococaine	0.216	0.223	0.547	6.214	1.415	0.539
Allylescaline	0.763	0.294	0.653	0.853	0.255	1.084
a-Naphthoflavone	0.465	0.291	0.921	10.473	0.377	0.565
Androsta-1,4-dien-3-one, 4-chloro-6,17-dihydroxy-17-methyl-, (6a,17a)-	0.037	0.384	0.004	0.637	0.867	1.450
Anilofos	0.532	0.400	0.637	2.174	1.913	0.503
Anthracene, 2-methyl-	0.417	0.006	0.135	0.872	0.828	1.190
Anthranilic acid	0.944	0.072	0.281	1.011	1.758	1.130
Arabinofuranose, 1,2,3,5-tetrakis-O-(trimethylsilyl)-	0.013	0.014	0.340	0.411	0.351	1.260
Arachidic acid	0.762	0.020	0.411	1.048	0.734	1.109
Arachidonic acid	0.080	0.583	0.260	4.464	2.983	0.527
Arsenous acid, tris(trimethylsilyl) ester	0.134	0.161	0.052	0.103	2.969	24.915
a-Tocopherol	0.502	0.111	0.702	0.306	0.220	2.088
Azatadine	0.001	0.006	0.001	5.428	6.244	0.124
Azetidine, 1-acetyl-2-methyl-	0.002	0.327	0.039	0.663	0.832	1.410
Aziridine, 1-ethenyl-	0.034	0.049	0.102	0.149	0.265	1.816
Behenic acid	0.673	0.019	0.561	1.057	0.661	1.090
Benzaldehyde	0.048	0.703	0.108	0.726	0.941	1.753
Benzaldehyde, 2,5-dimethoxy-	0.391	0.001	0.023	0.796	0.310	1.275
Benzaldehyde, 2-methyl-	0.638	0.555	0.216	0.932	0.882	1.593
Benzaldehyde, 3,4-dimethyl-	0.053	0.066	0.028	0.696	0.770	1.391
Benzenamine, 4-bromo-2,6-dimethyl-	0.101	0.008	0.075	0.719	0.458	1.419
Benzene, (1-ethylundecyl)-	0.152	0.086	0.114	0.347	0.410	1.774
Benzene, (1-methyldecyl)-	0.271	0.850	0.080	0.690	0.989	1.502
Benzene, (1-methyldodecyl)-	0.026	0.829	0.052	0.403	1.103	1.895
Benzene, 1,1'-(1,2-cyclobutanediyl)bis-, trans-	0.495	0.974	0.432	0.810	0.975	1.278
Benzene, 1,1'-(1-methyl-1,3-propanediyl)bis-	0.359	0.231	0.271	1.224	1.679	0.652

Benzene, 1,3-bis(1,1-dimethylethyl)-	0.159	0.103	0.040	0.827	0.823	1.304
Benzene, 1,3-diethyl-	0.418	0.361	0.954	1.290	0.872	0.957
Benzene, 1,4-bis(1-methylethyl)-	0.277	0.410	0.449	0.166	1.363	2.517
Benzene, 1-acetoxy-2,6-dimethoxy-4-(2-propenyl)-	0.283	0.001	0.448	0.443	0.002	1.512
Benzeneacetic acid	0.035	0.125	0.102	0.694	0.832	1.232
Benzenepropanoic acid, 3,5-bis(1,1-dimethylethyl)-4-hydroxy-, methyl ester	0.024	0.004	0.080	0.543	0.388	1.557
Benzenesulfonamide, N-butyl-	0.981	0.036	0.222	1.179	3.803	2.870
Benzimidazole, 5-trifluoromethyl-2-methyl-	0.246	0.142	0.105	0.854	0.860	1.199
Benzo[b]dioxan, 4,4-dimethyl-5-nitro-3-methoxy-	0.003	0.003	0.796	0.075	0.078	0.755
Benzoic acid, 2-hydroxy-3-[(2-hydroxy-4-methoxy-6-propylbenzoyl)oxy]-4-methoxy-6-propyl-, methyl ester	0.548	0.485	0.375	1.084	1.077	1.124
benzoic acid, 3,5-dimethyl-, trimethylsilyl ester	0.002	2.89E-05	0.004	0.035	0.090	1.970
Benzoic acid, 4-ethoxy-, ethyl ester	0.082	0.091	0.015	0.828	0.836	1.303
Benzoic acid, methyl ester	0.120	0.126	0.078	0.843	0.830	1.239
Benzophenone	0.481	0.573	0.039	0.873	0.940	1.292
Boric acid	0.910	0.866	0.685	0.998	0.972	0.904
Butanedioic acid	0.699	0.044	0.027	1.035	2.529	1.242
Butanoic acid, 2,4-bis[(trimethylsilyl)oxy]-, trimethylsilyl ester	0.063	0.050	0.202	1.342	1.465	1.674
Butanoic acid, 2-methyl-3-[(trimethylsilyl)oxy]-, trimethylsilyl ester	0.400	0.668	0.280	0.885	1.037	1.655
Butanoic acid, 3,4-bis[(trimethylsilyl)oxy]-, trimethylsilyl ester	0.545	0.463	0.197	1.114	1.152	1.406
Butylated Hydroxytoluene	0.407	0.147	0.922	1.500	1.850	1.106
Butylated Hydroxytoluene	0.687	0.261	0.248	0.934	0.828	1.200
Campesterol	0.398	0.023	0.767	0.725	0.556	1.071
Carbazole	0.417	0.461	0.239	1.802	1.926	0.074
Carbonic acid, hexyl prop-1-en-2-yl ester	0.529	0.317	0.862	1.130	1.588	1.206
Cardamonin, bis(tert-butyldimethylsilyl) ether	0.118	0.044	0.656	0.508	0.242	1.063
c-Dodecalactone	0.027	0.006	0.730	11.390	23.549	0.988
Cetene	0.062	0.166	0.319	0.234	3.199	2.102
Cholest-3,5-diene	0.985	0.323	0.987	1.175	0.341	0.846

Cholest-4-en-3-ol, (3a)-	0.305	0.715	0.976	1.671	0.816	1.178
Cholest-5-en-3-ol (3a)-, carbonochloridate	0.339	0.002	0.226	6.760	65.767	0.020
Cholest-7-en-3-ol, (3a,5a)-	0.186	0.004	0.674	0.752	0.411	1.089
Cholesta-2,4-diene	0.476	0.086	0.614	0.920	0.213	1.005
Cholesta-3,5-dien-7-one	0.202	0.350	0.618	2.839	1.667	2.246
Cholesta-3,5-diene	0.284	0.094	0.470	0.470	0.297	1.702
Cholesta-4,6-dien-3-ol, (3a)-	0.479	0.206	0.204	1.802	6.908	0.024
Cholestan-3-ol, (3a,5a)-	0.499	0.177	0.334	0.893	0.446	1.159
Cholestane-3,5-diol, 5-acetate, (3a,5a)-	0.320	0.474	0.548	6.763	2.560	4.221
Cholesterol	0.621	0.992	0.822	1.169	1.034	1.005
Cholesterol	0.493	0.877	0.853	0.798	1.137	0.948
Cholesteryl benzoate	0.534	0.576	0.293	119.810	53.567	0.041
Cicaprost	0.666	0.268	0.288	0.417	0.154	5.403
cis-.DELTA.2-11-Methyldodecenoic acid	0.066	0.226	0.894	0.456	0.619	1.039
Cysteine	0.519	0.325	0.779	1.918	0.341	1.370
Cytosine	0.222	0.197	0.281	1.607	3.249	1.965
D-(-)-Fructofuranose, pentakis(trimethylsilyl) ether (isomer 2+1)	0.091	0.031	0.077	0.261	0.029	3.780
D-(-)-Fructopyranose (isomer 1)	0.104	0.781	0.377	0.205	0.727	2.348
D-(-)-Tagatofuranose, pentakis(trimethylsilyl) ether (isomer 1)	0.247	0.506	0.593	38.809	23.393	0.060
D-Alanine, N-ethoxycarbonyl-, octadecyl ester	0.442	0.217	0.794	0.426	0.085	1.798
D-Allofuranose, pentakis(trimethylsilyl) ether	0.641	0.400	0.316	1.085	1.114	1.181
Decanoic acid	0.989	0.003	0.101	1.870	2.622	1.265
Decyl heptyl ether	0.455	0.260	0.220	0.783	0.719	1.686
Dehydroabietic acid	0.142	0.067	0.742	1.308	0.685	1.073
D-Erythro-Pentofuranose, 2-deoxy-1,3-bis-O-(trimethylsilyl)-, bis(trimethylsilyl) phosphate	0.226	0.003	0.082	2.291	0.059	0.670
Desmosterol	0.594	0.082	0.198	1.093	0.635	1.348
D-Fructose	0.709	0.529	0.965	1.077	0.632	1.222
D-Galactose	0.659	0.433	0.872	0.586	0.396	1.423
D-Glucoheptose	0.808	0.030	0.215	1.090	0.421	1.388
D-Glucose	0.006	0.149	0.746	0.344	0.503	0.935

Dichloroacetic acid, 2-pentadecyl ester	0.762	0.102	0.669	1.051	0.336	0.803
Dichloroacetic acid, tetradecyl ester	0.518	0.142	0.246	0.712	0.388	2.138
Diglycolic acid	0.020	0.835	0.312	0.052	0.546	3.409
DL-3-Aminoisobutyric acid	0.163	0.052	0.725	3.785	33.262	0.813
DL-3-Methyl-2-butanol	0.309	0.923	0.719	0.056	0.106	5.867
D-Leucine	0.636	0.168	0.911	0.313	0.036	2.139
DL-Norleucine	0.111	0.183	0.899	0.191	0.026	2.675
DL-Ornithine	0.005	4.74E-05	0.242	23.728	8.147	0.018
DL-Phenylalanine	0.411	0.017	0.915	1.210	1.895	1.033
DL-Valine	0.088	0.087	0.009	0.279	0.320	12.451
Doconexent	0.166	0.181	0.228	1.370	1.231	1.239
Dodecanoic acid, isooctyl ester	0.352	0.950	0.457	0.423	0.636	0.845
Dodecanoic acid	0.096	0.053	0.219	1.386	1.308	1.234
Erythrono-1,4-lactone, (Z)-	0.016	0.562	0.173	1.764	0.950	1.254
Ethanamine	0.435	0.701	0.296	0.690	0.822	1.606
Ethane, isocyanato-	0.958	0.323	0.406	1.690	0.103	2.655
Ethanol, 2-(trimethylsilyl)-	0.081	0.027	0.001	71.069	39.236	0.015
Ethanone, 1-(2-pyridinyl)-	0.092	0.006	0.134	0.750	0.082	1.321
Ethanone, 2-(3-chlorophenyl)-1-(1-pentyl-1H-indol-3-yl)-	0.169	6.81E-06	0.334	0.557	0.132	1.172
Ethoxyacetic acid	0.886	0.025	0.620	1.116	1.859	0.849
Ethyl 1-methylpipecolinate	0.006	0.006	0.008	1.941	1.923	1.692
Ethyl 9-hexadecenoate	0.296	0.500	0.993	0.380	1.774	2.199
Glucuronolactone, trisO-(trimethylsilyl)-	0.097	0.004	0.316	0.638	0.031	0.862
Glyceric acid	0.877	0.033	0.244	1.020	1.370	1.773
Glycerol monostearate	0.009	0.054	0.089	1.731	11.812	1.372
Glycerol tricaprylate	0.249	0.251	0.222	0.366	7.197	2.870
Glycerol, 1,2-di(TMS)-	0.069	0.186	0.153	0.112	0.675	2.888
Glycerol	0.832	0.144	0.344	1.050	0.554	1.116
Glycine, di-TMS	0.537	0.625	0.299	3.130	2.073	6.908
Glycitin	0.954	0.978	0.079	1.644	1.411	524.700
Glycol salicylate	0.407	0.554	0.177	0.804	0.869	2.247
Glycolic acid	0.101	0.992	0.963	1.512	0.981	1.028

Guanidine, N,N-dimethyl-	0.344	0.942	0.132	0.879	1.004	1.215
Heptadecanoic acid, glycerine-(1)-monoester, bis-O-trimethylsilyl-	0.008	0.816	0.137	1.897	4.144	1.327
Heptadecanoic acid	0.050	0.131	0.177	1.394	1.222	1.199
Heptanoic acid	0.767	0.001	0.552	0.976	1.296	1.090
Hexadecanoic acid, 4-[(trimethylsilyl)oxy]butyl ester	0.012	0.726	0.065	0.551	1.846	1.733
Hexadecanoic acid, ethyl ester	0.324	0.592	0.480	1.528	0.444	0.868
Hexadecanoic acid, methyl ester	0.560	0.538	0.180	0.860	1.750	2.231
Hexahydroindole	0.022	0.047	0.058	0.671	0.684	1.377
Hexanedioic acid	0.761	0.300	0.304	0.881	1.296	1.741
Hexanedioic acid, mono(2-ethylhexyl)ester	0.693	0.287	0.250	1.051	1.157	1.185
Hexanoic acid	0.341	0.089	0.341	1.202	1.194	0.919
Hexatriacontane	0.093	0.607	0.483	3.595	0.456	0.825
Hippuric acid	0.024	0.007	0.186	0.647	0.539	1.178
Hydracrylic acid	0.976	0.423	0.150	0.991	0.917	1.249
Hymexazole, tert-butyldimethylsilyl ether	0.064	0.093	0.220	1.187	1.326	1.434
Indole-3-lactic acid	0.152	8.86E-05	0.159	1.406	9.735	0.797
Inosose-2, 1,3,4,5,6-pentakis-O-(trimethylsilyl)-, myo-	0.002	0.001	0.044	2.368	3.103	0.622
Isobornyl acetate	0.591	0.531	0.203	0.923	1.411	2.234
Isoflavone-7-O-a-D-glucopyranoside	0.868	0.412	0.389	0.676	0.142	3.065
Isopropyl myristate	0.875	0.569	0.983	0.837	0.207	0.627
Itaconic acid	0.991	0.602	0.919	0.249	21.247	0.755
Itaconic acid	0.122	0.142	0.997	0.428	0.827	0.877
L-3-Methylhistidine	0.691	0.397	0.574	1.770	0.361	0.743
L-5-Oxoproline	0.414	0.352	0.191	0.878	0.877	1.224
Labda-8(20), 13-dien-15,19-dioic acid-di(trimethylsilyl) ester	0.842	0.036	0.799	0.902	0.445	1.122
Lactic Acid	0.362	0.349	0.928	1.550	1.583	1.571
L-Alanine	0.327	0.420	0.670	1.790	0.791	0.838
Lanopalmitic acid, 2-O-TMS	0.243	0.010	0.307	0.799	0.524	1.198
L-Arginine	0.251	0.482	0.497	0.366	1.750	0.232
L-Aspartic acid	0.509	0.042	0.024	1.405	9.524	2.360

L-Aspartic acid	0.358	0.456	0.154	1.467	1.152	1.233
L-Cysteine	0.706	0.876	0.663	0.909	0.743	1.067
Levulinic acid	0.256	0.129	0.044	0.852	0.838	1.255
L-Fucose	0.410	0.222	0.086	1.802	26.018	1.300
L-Glutamic acid	0.212	0.582	0.947	4.409	1.093	0.780
L-Glutamic acid, bis(trimethylsilyl) ester	0.014	0.022	0.760	2.120	2.055	0.975
L-Hydroxyproline, (E)-	0.127	0.060	0.514	3.331	5.316	0.932
Lignoceric acid	0.359	0.025	0.477	1.144	0.670	1.107
L-Isoleucine	0.914	0.615	0.823	1.059	0.889	1.053
L-Kynurenine	0.826	0.310	0.177	0.852	0.377	0.610
L-Leucine	0.330	0.381	0.189	0.646	0.838	1.312
L-Lysine	0.186	0.129	0.331	1.474	0.530	0.819
L-Lysine, N6,N6-bis(trimethylsilyl)-, trimethylsilyl ester	0.177	0.001	0.419	0.442	0.008	1.122
L-Methionine	0.205	0.058	0.261	2.040	5.676	1.494
l-Norvaline, N-ethoxycarbonyl-, heptyl ester	0.194	0.096	0.720	2.583	4.217	0.321
L-Ornithine	0.001	0.002	0.520	2.351	2.044	0.814
L-Phenylalanine	0.130	0.111	0.001	0.414	2.388	8.546
L-Proline	0.883	0.030	0.448	2.042	2.494	0.570
L-Proline, trimethylsilyl ester	0.206	0.052	0.023	0.272	0.089	25.204
L-Serine	0.474	0.299	0.272	1.112	0.840	2.134
L-Threitol	0.144	0.026	0.038	1.381	1.422	1.412
L-Threonine	0.453	0.024	0.061	1.696	1.701	5.315
L-Tryptophan, 1-(trimethylsilyl)-, trimethylsilyl ester	0.712	0.001	0.042	1.158	0.120	0.489
L-Tyrosine	0.405	0.001	0.254	0.837	0.027	1.079
L-Valine	0.261	0.607	0.240	5.372	1.310	2.868
Lys-Gly-Asp-Ser	0.034	0.016	0.434	0.020	0.014	1.650
Malic acid	0.009	0.027	0.396	2.513	2.136	0.877
Mandelonitrile	0.012	0.012	0.056	7.201	6.823	0.338
m-Cresol	0.044	0.016	0.021	0.351	0.592	1.350
Menthol, 1'-(butyn-3-one-1-yl)-, (1S,2S,5R)-	0.489	0.904	0.995	0.033	3.780	0.629
Mercaptoacetic acid	0.109	0.211	0.976	2.176	1.667	0.947
meso-Erythritol	0.914	0.440	0.386	1.053	1.103	1.132

Methyl (3,4-dimethoxyphenyl)(hydroxy)acetate	0.635	0.079	0.061	0.915	0.507	1.442
Methyl 4-methoxysalicylate	0.056	0.187	0.001	0.250	0.406	9.931
Methyl dehydroabietate	0.760	0.031	0.972	0.435	0.016	1.790
Methyl galactoside (1S,2S,3S,4R,5R)-	0.116	0.166	0.232	3.040	4.666	0.318
Methylmalonic monoamide, O,O'-bis(trimethylsilyl)-	0.046	0.691	0.022	1.559	1.122	2.258
Methylsuccinic acid	0.307	0.226	0.078	1.085	1.172	1.148
Met-Val	0.436	0.223	0.002	1.802	1.225	0.123
Mildronate	0.642	0.001	0.775	8.984	254.320	0.324
Monomethyl succinate, trimethylsilyl ester	0.011	0.003	0.006	0.672	0.704	1.489
m-Toluic acid	0.832	0.031	0.263	1.083	1.166	1.109
Myristic acid	0.989	0.731	0.116	1.002	0.964	1.206
N-((Dimethylamino)methylene)-2,2,2-trifluoroacetamide	0.026	0.782	0.798	1.444	1.323	1.043
N-(2,4-Dinitrophenyl)-L-methionine	0.257	0.013	0.574	0.421	0.046	1.366
N,N-Diethylaniline	0.164	0.176	0.047	0.825	0.860	1.306
Na-(tert-Butoxycarbonyl)-L-aspartic acid	0.008	0.583	0.008	0.587	0.914	1.735
N-a-(tert-Butoxycarbonyl)-L-lysine	0.696	0.902	0.688	0.339	0.346	1.089
Na,Na-Dimethyllysine, methyl ester	0.510	0.161	0.290	2.240	2.084	3.169
N-Acetyl-D-galactosamine, (isomer 2)	0.068	0.007	0.799	0.659	0.446	1.056
N-Acetyl-L-alanine	0.502	0.041	0.136	0.844	23.964	2.317
N-Acetyl-L-carnosine	0.032	0.123	0.010	0.690	0.871	1.948
N-Acetyl-S-benzyl-L-cysteine	0.750	0.175	0.303	0.698	0.036	4.805
Naled	0.021	0.223	0.210	24.370	1.143	0.083
Naphthalene	0.033	0.138	0.140	0.673	0.666	1.229
Naphthalene, 2-ethenyl-	0.221	0.365	0.193	0.876	0.919	1.168
n-Dodecylamine	0.590	0.223	0.155	2.174	1.539	0.348
Niacin	0.656	0.131	0.043	1.082	4.398	1.546
Niacinamide	0.010	0.049	0.152	0.466	0.216	1.227
N-Isobutyrylglycine	0.205	0.013	0.086	1.340	17.081	1.424
N-Methyl-L-proline, tert.-butyldimethylsilyl ester	0.004	0.075	0.225	28.576	14.313	0.317
Nonadecanoic acid	0.120	0.502	0.097	1.340	0.871	1.567
Nonanoic acid	0.530	0.005	0.243	1.089	1.494	1.131

Octanal, 2-(phenylmethylene)-	0.063	1.000	0.056	0.219	1.044	4.791
Octanoic acid	0.629	0.436	0.490	0.930	1.116	1.094
Oleic Acid, (Z)-	0.467	2.74E-05	0.086	1.875	4.947	1.449
O-Methyl-N,N'-diisopropylisourea	0.203	0.205	0.175	10.710	34.623	0.165
Ornithine	0.400	0.029	0.261	13.715	25.428	9.852
Oxalic acid	0.819	0.570	0.276	1.015	0.892	1.763
Oxalic acid, di(cyclohexylmethyl) ester	0.882	0.635	0.511	0.825	0.380	0.497
Oxazole, 2-(8Z)-8-heptadecen-1-yl-4,5-dihydro-	0.450	0.015	0.186	1.128	0.600	1.186
Palmitelaidic acid	0.307	0.209	0.104	1.141	1.125	1.217
Palmitic acid vinyl ester	0.323	0.386	0.735	1.623	0.480	1.043
Palmitic Acid	0.927	0.549	0.501	0.980	0.806	1.172
Palmitoleamide	0.565	0.228	0.088	0.902	0.718	1.548
p-Cresol	3.22E-05	2.21E-05	0.016	0.152	0.157	8.023
Pentadecanoic acid	0.484	0.830	0.084	1.104	0.967	1.318
Pentanedioic acid	0.011	0.005	0.100	0.414	0.436	1.409
Pentanoic acid	0.816	0.046	0.630	1.469	1.886	0.645
Pentitol, 3-desoxy-tetrakis-O-(trimethylsilyl)-	0.160	0.274	0.211	1.249	1.166	1.189
Petroselinic acid	0.710	0.744	0.997	0.240	0.324	3.234
Phenol, 3-(ethylamino)-4-methyl-	0.057	0.811	0.035	6.552	2.864	0.177
Phenol	0.258	0.210	0.226	0.854	0.779	1.147
Phenylalanine	0.528	0.837	0.928	1.945	1.093	0.891
Phenylpropanolamine	0.208	0.004	0.348	0.618	0.541	1.330
Phloretic acid	0.193	0.189	0.200	3.261	4.052	0.324
Phosphoric acid, bis(trimethylsilyl)monomethyl ester	0.547	0.213	0.512	0.882	0.647	1.141
p-Hydroxydiisopropylbenzene	0.509	0.070	0.437	1.175	1.323	0.884
Picolinic acid	0.003	0.002	0.864	1.970	2.290	0.918
Pimelic acid	0.083	0.007	0.247	1.345	1.858	0.837
Pipecolic acid	0.052	0.194	0.655	26.372	30.081	0.969
Propanedioic acid	0.493	0.682	0.198	1.080	1.136	1.264
Propanoic acid, 2-methyl-, hexyl ester	0.213	0.015	0.199	0.700	0.526	1.507
p-Toluic acid	0.004	0.003	0.857	31.105	38.377	1.113

Pyrazole-4-carboxaldehyde-, 3,5-dimethyl-1-(3,4-dimethylphenyl)-	0.575	0.210	0.174	0.933	0.863	1.148
Pyrazolo[3,4-b]pyridin-6-one, 1,7-dihydro-1,3,4-trimethyl-	0.711	0.029	0.247	1.377	0.141	0.772
Pyroglutamic acid	0.099	0.128	0.269	1.568	1.799	0.770
Pyrrole-2-carboxylic acid	0.038	0.049	0.139	4.085	3.222	0.419
Ribitol	0.333	0.686	0.592	0.879	0.956	1.071
Ribonic acid, 2,3,4,5-tetrakis-O-(trimethylsilyl)-, trimethylsilyl ester	0.521	0.468	0.250	0.872	0.869	1.332
Sarcosine	0.731	0.673	0.766	0.661	0.639	1.423
S-Carboxymethyl-L-cysteine	0.535	0.001	0.086	1.875	8.018	1.186
Ser-Arg	0.252	0.902	0.577	3.723	2.309	0.253
Serine	0.798	0.191	1.21E-05	2.297	0.832	10.125
Silicic acid, diethyl bis(trimethylsilyl) ester	0.232	0.425	0.207	0.313	0.488	2.818
Stearic acid	0.008	0.008	0.094	0.226	0.373	2.530
Stigmast-5-ene, 3a-(trimethylsiloxy)-, (24S)-	0.632	0.041	0.158	1.072	0.671	1.383
Succinamide	0.132	0.183	0.270	0.254	0.280	1.925
Sulfide, bis(2-cyano-3,4-dihydro-2,3,3-trimethyl-2H-pyrrol-5-yl)-	0.190	0.107	0.303	0.056	0.205	0.551
Sulfuric acid	0.837	0.670	0.790	1.098	0.835	1.113
Sulfurous acid, 2-ethylhexyl pentyl ester	0.549	0.305	0.218	1.555	0.392	1.864
Sulfurous acid, octyl 2-propyl ester	0.752	0.494	0.682	0.959	1.067	0.917
Supraene	0.006	0.329	0.025	0.225	0.772	3.008
Syringaldehyde	0.334	0.968	0.428	1.160	0.996	1.112
Talose	0.093	0.191	0.628	1.486	1.278	1.110
Tartaric acid	0.033	0.040	0.315	0.648	0.424	1.324
Tartronic acid	0.190	0.321	0.876	3.194	1.998	1.485
Tetradecanoic acid, ethyl ester	0.092	0.606	0.085	0.121	0.762	8.708
Tetrahydrogeranyl formate	0.278	0.237	0.103	0.832	0.837	1.310
Thiazolidine-2-carboxylic acid	0.302	0.001	0.235	1.365	3.489	1.647
Thieno[2,3-d]pyrimidine, 4-[4-(4,5-dihydro-5,5-dimethyl-2-thiazolyl)-1-piperazinyl]-6-propyl-	0.174	0.321	0.341	1.406	0.910	1.130
Thiomiltefosine	0.307	0.827	0.938	0.366	0.874	0.944
Thymidine	0.917	0.057	0.008	1.038	0.281	1.770

Timonacic	0.488	0.432	0.365	0.791	1.162	0.829
trans-4-Oxo-2-pentenoic acid	0.015	0.243	0.025	0.763	0.892	1.332
Triethanolamine	0.586	0.906	0.199	0.890	0.991	1.490
Trimellitic acid anhydride	0.916	0.212	0.868	2.836	0.009	0.570
Trimethylsilyl 2-(2-(2-(trimethylsilyloxy)ethoxy)ethoxy)acetate	0.310	0.811	0.907	1.251	1.019	1.032
Trimethylsilyl 2-amino-4-oxo-4-pyrrol2-[(trimethylsilyl)amino]phenylmorphobutanoate	0.184	0.223	0.151	8.959	1.243	0.317
Trimethylsilyl 3-((trimethylsilyl)thio)propanoate	0.365	0.445	0.708	0.692	0.769	1.075
Trisaminol, 3-OTMS	0.521	0.064	0.494	0.797	0.235	1.334
Tyrosine	0.443	0.572	0.446	0.772	0.835	1.239
Tyrosol	0.800	0.396	0.779	1.099	0.664	0.859
Uracil	0.550	0.008	0.002	14.436	9.244	3.612
Urea	0.614	0.519	0.252	1.061	0.928	0.571
Urea	0.060	0.528	0.473	0.797	1.423	1.207
Urea, N,N-dimethyl-N'-butyl-N'-propyl-	0.363	0.797	0.074	0.895	0.974	1.240
Urea, triethyl-	0.178	0.210	0.651	0.045	0.047	3.457
Uridine	0.941	0.003	0.759	1.027	0.115	1.052
Valproic acid	0.997	0.078	0.711	5.728	0.180	0.199
Vamidothion	0.403	0.253	0.479	0.668	1.419	1.418
Yangonin	0.460	0.134	0.009	0.998	0.498	14.547
